# Effectiveness of physical activity interventions on undergraduate students’ mental health: systematic review and meta-analysis

**DOI:** 10.1093/heapro/daae054

**Published:** 2024-06-25

**Authors:** Kevin Huang, Emma M Beckman, Norman Ng, Genevieve A Dingle, Rong Han, Kari James, Elisabeth Winkler, Michalis Stylianou, Sjaan R Gomersall

**Affiliations:** Health and Wellbeing Centre for Research Innovation, School of Human Movement and Nutrition Sciences, The University of Queensland, St Lucia, Queensland 4072, Australia; School of Human Movement and Nutrition Sciences, The University of Queensland, St Lucia, Queensland 4072, Australia; School of Human Movement and Nutrition Sciences, The University of Queensland, St Lucia, Queensland 4072, Australia; School of Psychology, The University of Queensland, St Lucia, Queensland 4072, Australia; School of Psychology, The University of Queensland, St Lucia, Queensland 4072, Australia; School of Psychology, The University of Queensland, St Lucia, Queensland 4072, Australia; School of Human Movement and Nutrition Sciences, The University of Queensland, St Lucia, Queensland 4072, Australia; School of Human Movement and Nutrition Sciences, The University of Queensland, St Lucia, Queensland 4072, Australia; Health and Wellbeing Centre for Research Innovation, School of Human Movement and Nutrition Sciences, The University of Queensland, St Lucia, Queensland 4072, Australia; School of Health and Rehabilitation Sciences, The University of Queensland, St Lucia, Queensland 4072, Australia

**Keywords:** physical activity, mental health, university students, health behaviours, systematic review

## Abstract

This systematic review and meta-analysis assessed the effectiveness of physical activity interventions on undergraduate students’ mental health. Seven databases were searched and a total of 59 studies were included. Studies with a comparable control group were meta-analysed, and remaining studies were narratively synthesized. The included studies scored very low GRADE and had a high risk of bias. Meta-analyses indicated physical activity interventions are effective in reducing symptoms of anxiety (*n* = 20, standardized mean difference (SMD) = −0.88, 95% CI [−1.23, −0.52]), depression (*n* = 14, SMD = −0.73, 95% CI [−1.00, −0.47]) and stress (*n* = 10, SMD = −0.61, 95% CI [−0.94, −0.28]); however, there was considerable heterogeneity (anxiety, *I*^*2*^ = 90.29%; depression *I*^*2*^ = 49.66%; stress *I*^*2*^ = 86.97%). The narrative synthesis had mixed findings. Only five studies reported being informed by a behavioural change theory and only 30 reported intervention fidelity. Our review provides evidence supporting the potential of physical activity interventions in enhancing the mental health of undergraduate students. More robust intervention design and implementation are required to better understand the effectiveness of PA interventions on mental health outcomes.

**Contribution to Health Promotion**

- Physical activity is a known health behaviour that protects undergraduate students’ mental health, but the exact effects were unknown.
- Meta-analyses identified that physical activity had positive effects on stress, anxiety and depression. Narrative synthesis found mixed results.
- The syntheses found high levels of variability, such as the type of physical activity and duration of intervention.
- The studies were of low quality and certainty of evidence as marked by an under-reporting of intervention fidelity and potential under-utilization of behaviour change theories.
- Physical activity could be a mental health promotion strategy, but future interventions need to be better reported and grounded in theory.

## BACKGROUND

Undergraduate students starting university face numerous challenges. For many students, this significant life transition includes moving to a new city or state and away from sources of support typically found in friends and family (Meehan and Howells, 2019). While newfound independence and autonomy are welcomed by some, the lack of emotional support (Bitsika *et al*., 2010), academic workload (Fernández *et al*., 2017) and the self-directed learning style adopted in many universities (Bayram and Bilgel, 2008) can result in significant challenges for students’ mental health and wellbeing. Undergraduate university students, typically in the 18–25 age range, face the highest risk of experiencing the first onset of mental health issues (Solmi *et al*., 2022), such as poor wellbeing and symptoms of psychological distress, including anxiety and depression. Furthermore, a significantly higher proportion of undergraduate students are diagnosed with some form of mental health illness relative to individuals of other ages (Solmi *et al*., 2022) and this population also tends to report higher levels of psychological distress when compared with non-students in three national surveys (Cvetkovski *et al*., 2012).

Globally, there has been an increase in the proportion of undergraduate students experiencing poor mental health (Lipson *et al*., 2022). For instance, a longitudinal study conducted between 2013 and 2021 across the United States showed a 50% increase in mental health diagnoses and a two-fold increase in displaying poor mental health symptoms in university students (Lipson *et al*., 2022). Mental health services have not been able to meet the increased demand, resulting in long wait times and inadequate access to psychologists, psychiatrists and other mental health professionals (Broglia *et al*., 2018; Dingle *et al*., 2021; Punton *et al*., 2022). Given the escalating need and significant barriers to accessing care, there is a key imperative to examine prevention strategies for mental health in undergraduate students.

Participation in physical activity (PA; WHO, 2023) is one strategy that has been shown to improve physical health (Benaich *et al*., 2021) and prevent poor mental health (Román-Mata *et al*., 2020). Participating in a minimum of 150 min per week of moderate to vigorous intensity PA is protective against symptoms of depression (Peluso and Guerra de Andrade, 2005) and is associated with lower anxiety and stress, and a higher quality of life (Herbert *et al*., 2020). PA affects mental health in both direct ways (e.g. increasing endorphin release, which improves mood; Mikkelsen *et al*., 2017) and indirect ways, such as via improving the quality of sleep, which can lead to better mental health (Babaeer *et al*., 2020). Conversely, insufficient PA is associated with poorer mental health, increased self-harm and more suicide attempts in university students (Grasdalsmoen *et al*., 2020). Despite the health benefits of PA, however, undergraduate students remain insufficiently active (Mahony *et al*., 2019). For example, an Australian study found 30% fewer undergraduate students reported sufficient PA levels in 2020 compared to 2018 (Gallo *et al*., 2020). Another study conducted in 2022, which had a sample of more than 15 000 South East Asian undergraduate students, reported that 39.7% of their participants were physically inactive (Rahman *et al*., 2022).

While available evidence supports that PA can enhance mental health outcomes in undergraduate students (e.g. Mailey *et al*., 2010; Melnyk *et al*., 2014; Herbert *et al*., 2020), individual studies report mixed findings. For example, in a study with German students, a 6-week aerobic exercise intervention was found to reduce symptoms of depression and stress over time relative to a waitlist control condition, although the effect on anxiety was not significant (Herbert *et al*., 2020). However, in a study using a 2-month internet-delivered PA intervention for undergraduate students, Mailey *et al*. (2010) found that those in the intervention group reported a significant decrease in anxiety compared to the waitlist control group. Interestingly, Mailey *et al*. (2010) also found that depression decreased in both groups. One scoping review has attempted to synthesize available evidence in young people aged 12–26 years and concluded that PA interventions seemed to be effective in reducing certain symptoms of poor mental health, particularly with higher intensity PA interventions, but found varying degrees of effectiveness depending on intervention design, intensity and context (Pascoe *et al*., 2020). This review, however, focused on young adults more broadly and did not exclusively examine university students in their unique context, included a small number of studies and did not include a meta-analysis. Given that most existing studies on PA interventions targeting undergraduate students’ mental health have been synthesized narratively (Lubans *et al*., 2016; Dogra *et al*., 2018; Ellard *et al*., 2023; Donnelly *et al*., 2024), we have observed that there are currently no systematic reviews on this topic that have synthesized findings quantitatively, for example, using meta-analyses, which can offer additional insights for understanding effectiveness (Melendez-Torres *et al*., 2017; Mellor, 2021).

Synthesizing the literature that examines the impact of PA on mental health outcomes specifically in university students is warranted given the unique context of affordances and challenges associated with being a university student (Meehan and Howells, 2019). Such an endeavour can provide insights into the effectiveness of PA interventions in enhancing the mental health of this population, as well as into the design and implementation of identified interventions, which is a critical context to consider when examining their effectiveness. Information about whether and how the design of PA interventions has been informed by conceptual or theoretical frameworks that promote behaviour change (Michie *et al*., 2009) is particularly important as these facilitate the development, assessment and replicability of PA interventions, and provide a deeper understanding of underlying mechanisms that support effectiveness. Therefore, the current study aims to systematically review and meta-analyse available literature to determine the effectiveness of PA interventions on mental health outcomes in undergraduate students.

## METHOD

This systematic review was registered with the International Prospective Register of Systematic Reviews (PROSPERO) on 8 June 2022 (CRD42022320558) and follows the Preferred Reporting Items for Systematic Reviews and Meta-Analyses (PRISMA) guidelines (Page *et al*., 2021).

### Search strategy and study selection

An initial scoping search was conducted by the first reviewer (K.H.) to test and develop various keywords based on the PICO question. The finalized keywords were then confirmed by four reviewers (K.H., S.G., E.B. and G.D.). Subsequently, a search strategy was developed in PubMed with the assistance of an academic librarian and later adapted to an additional six databases (CINAHL, PsycINFO, Embase, Web of Science, SportsDiscus and Scopus). The search was initially conducted on 24 May 2022 and subsequently updated on 14 August 2023. Medical Subject Headings (MeSH) were used as search terms for mental health outcomes where appropriate, alongside keywords representing various types of PA, and different ways to describe undergraduate university students. Complete search terms for each database are found in Supplementary Appendix A.

The results of the systematic searches were imported into Covidence. Following duplicate removal, title and abstracts and subsequently, full-text articles were independently screened by two reviewers based on predetermined eligibility criteria. Inter-rater discrepancies at both screening stages were resolved via discussion and, where necessary, a third reviewer was brought in to reach a consensus.

### Eligibility criteria

Peer-reviewed studies reporting on original research and published in English were included if the following criteria were met: (i) participants were enrolled in undergraduate university programs; (ii) PA interventions were at least 4 weeks in duration (Lally *et al*., 2010) and were part of experimental randomized controlled trials (RCTs), longitudinal, cohort, pre-post and single-arm studies; (iii) comparators included waitlist controls, no-treatment control, other health behaviour comparison or no control (e.g. single-arm or pre-post studies); (iv) studies reported mental health outcomes including symptoms of psychological distress, such as anxiety and depression, loneliness or social isolation and stress. The exclusion criteria were: (i) undergraduate student samples that were recruited based on convenience, rather than having a rationale for recruiting undergraduate students specifically, and studies that included a mix of undergraduate and postgraduate students in their data; (ii) interventions that targeted multiple health behaviours (e.g. diet and PA, or nutrition and PA) in the same intervention arm; (iii) cross-sectional studies; (iv) studies that exclusively examined Profile of Mood States (Pollock *et al*., 1979) as they are indicators of transitory states rather than mental health symptoms. Additionally, grey literature, including theses and dissertations, were excluded.

### Data extraction

After the screening process, the following data were extracted: study information (authors, year and country of publication, study design), participant information (e.g. sample size), characteristics of the intervention (type of PA intervention, duration and frequency, inclusion of theoretical concepts), fidelity (e.g. attendance), primary and secondary outcomes and measurement time points. Statistical data extracted (when reported) included: pre- and post-mean and standard deviation (SD), group change scores and their SD, *p* values for between-group effects, within-group effects and group-by-time interaction effects and standardized effect sizes. Data extraction was performed by two reviewers and a third reviewer was involved in resolving discrepancies where necessary.

### Quality assessment

Quality assessment was conducted by two reviewers independently using the Effective Public Health Practice Project (EPHPP) Quality Assessment Tool from the McMaster Evidence Review and Synthesis Team (Thomas *et al*., 2004; Armijo-Olivo *et al*., 2012). The EPHPP tool is used to assess the quality of RCTs and cohort (pre-post) experimental trials. Assessments were based on six domains, with each domain requiring a rating of weak, moderate or strong: (i) selection bias; (ii) study design; (iii) confounders; (iv) blinding; (v) data collection method and (vi) attrition. The overall quality of each study was assessed based on the total number of weak ratings given per domain: weak (≥2 weak ratings), moderate (one weak rating) and strong (no weak ratings).

The overall certainty of the evidence was assessed using the Grading of Recommendations Assessment, Development and Evaluation Method (GRADE; Guyatt *et al*., 2011). GRADE was performed for each of the outcomes included in the meta-analysis.

### Data synthesis

Data were synthesized in two ways. RCTs and non-RCTs with comparable control groups were synthesized using a meta-analysis. For studies that were not included in the meta-analysis, a narrative synthesis was conducted using Cochrane’s synthesis without meta-analysis (SWiM) guidelines (Campbell *et al*., 2020).

#### Meta-analyses

Meta-analyses were performed using STATA version 18 (StataCorp, 2023). Only the studies that compared a PA intervention with an appropriate control group not receiving the relevant intervention (RCTs and non-RCTs included) were included for determining the impact on continuous measures of mental health outcomes. The meta-analyses used inputs such as SMD and its standard error. Most studies reported cross-sectional means and SDs, but few reported on change or intervention effects with confidence intervals, and many were not obtainable even after attempting to contact corresponding authors. Therefore, for the meta-analysis, *t*-tests of post-intervention mean, and SD were used to calculate mean differences and their error, divided by the pre-intervention pooled SD to create SMDs that could be compared despite the disparate measures across studies. Studies that had more than one PA intervention group were counted as separate studies, with standard errors corrected for non-independence when they involved a shared control group using the method outlined by Cates (2015). The random DerSimonian-Laird effects model (Dersimonian and Laird, 1986) was used due to expected heterogeneity in the studies, which was reported in as *I*^2^ and Cochrane’s *Q* test. Begg’s test was performed to explore potential small-study effects and publication bias (Begg and Mazumdar, 1994), with trim-and-fill analyses reported wherever there were suggestions of publication bias (Duval and Tweedie, 2000). ‘Leave-one-out’ sensitivity analyses were performed to determine whether any studies had a disproportionately positive effect on the conclusion.

#### Narrative synthesis

The narrative synthesis included: studies that did not meet the eligibility criteria for the meta-analysis; and studies from the meta-analysis that tested outcomes in addition to anxiety, depression and stress. To facilitate the narrative synthesis, relevant study outcomes (e.g. depression, stress, anxiety) were coded as ‘+’, ‘−’, ‘/’ and ‘*’, where ‘+’ represents a statistically significant effect that favours improvement in the outcome, ‘−’ represents a statistically significant effect that favours a decline in the outcome, ‘/’ represents no statistical effect and ‘*’ represents outcomes that were tested using within groups statistical tests only, or where there was no statistical testing on the outcome (but pre-post descriptive data were reported).

## RESULTS

**Fig. 1:**
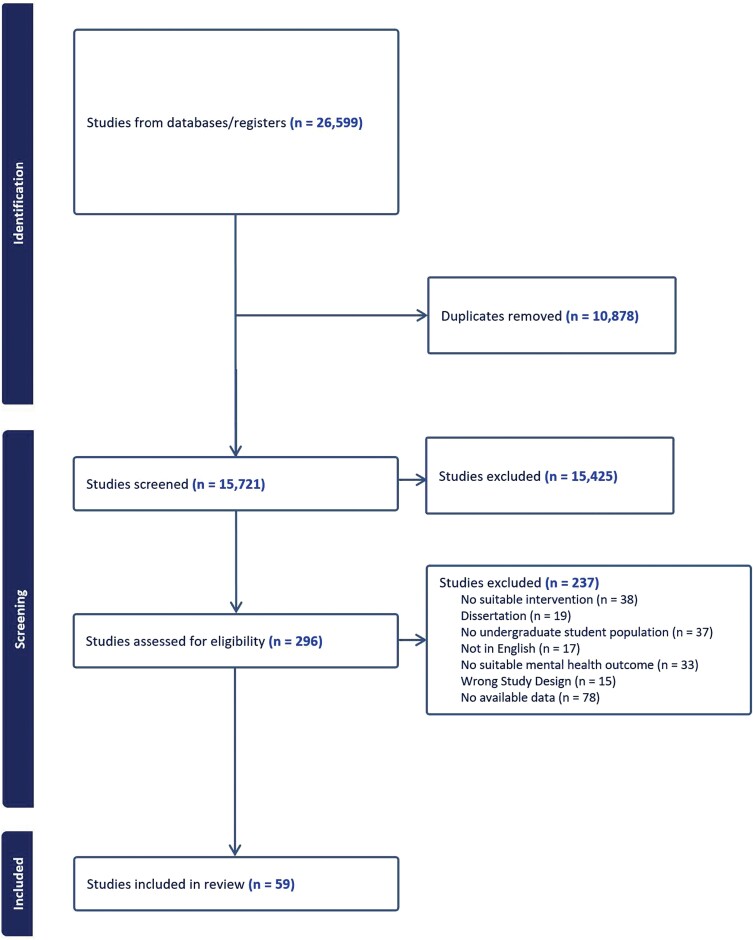
PRISMA Flowchart of study selection (Page *et al.*, 2021).

The study selection process is summarized in Figure 1 based on PRISMA guidelines. After title/abstract and full-text screening, 59 studies were deemed eligible for inclusion in the review, 38 of which were RCTs and 21 of which used other study designs. A total of 25 studies, comprising a mix of both RCTs and non-RCTs, were included in meta-analyses for the outcomes of anxiety, depression and stress.

### Quality assessment of studies

Based on the EPHPP Quality Assessment Tool, the quality of included studies was mostly weak (47 out of 59 studies). A summary of quality assessment results showed that about half of the studies received an overall rating of weak because of selection bias, poor study design (e.g. no control group), lack of controlling for potential confounders and/or blinding (see Supplementary Material B, Figure S3 for quality assessment breakdown). The certainty of evidence (GRADE) was very low for each of the outcomes in the meta-analysis (anxiety, depression and stress) as these studies were assessed as demonstrating a high risk of bias (limitation), heterogeneity, indirectness between included studies and imprecision (see Supplementary Material A, Table S3 for GRADE assessment).

### Study characteristics

Table 1 summarizes the main characteristics of the included studies and interventions. Aerobic exercise was the most reported PA intervention; 26 out of 59 studies implemented some form of aerobic exercise such as running, dancing or walking. Seventeen studies were mindfulness-based, low-intensity or low-impact exercises like yoga, baduanjin and taichiquan. Eight studies included resistance training such as muscular strengthening exercises, with six studies exposing participants to both aerobic and strength exercises. Two studies conducted pilates interventions, one used walking and another conducted an educational intervention only. The specific modalities for each study can be found in Table 2. Anxiety (*N* = 28), depression (*N* = 25) and stress (*N* = 21) were the most frequently assessed mental health outcomes across all included studies, with other outcomes being assessed in six or fewer studies each, and thus were selected to be meta-analysed. Commonly used measures were the State-Trait Anxiety Inventory (Spielberger *et al*., 1968), the Beck’s Depression Inventory (Beck et al., 1961) and the Perceived Stress Scale (Cohen *et al.*, 1983). Eight studies reported change in PA with the intervention, and of these, seven reported positive changes in PA and one reported post-intervention follow-up of PA as a measure of sustained improvement in PA behaviour (McFadden et al., 2017). It is worth noting that four studies were conducted during COVID-19 (BARĞI, 2022; Hamed et al., 2021; Terzioğlu et al., 2022; Tomar et al., 2023) and thus the mental health state of the participants might have been influenced by COVID-19-related factors.

**Table 1:** Summary of Study and Intervention Characteristics

Characteristic	Category	*N*	%
Country	Australia	2	3.39
	Canada	4	6.78
	China	16	27.11
	Germany	1	1.69
	Iran	4	6.78
	Japan	1	1.69
	Jordan	1	1.69
	Pakistan	1	1.69
	Saudi Arabia	2	3.39
	South Korea	2	3.39
	Spain	2	3.39
	The Netherlands	1	1.69
	Turkey	7	11.86
	United States	15	25.42
Study design	Randomized	35	40.68
	Non-randomized	24	59.32
Sample size	≥100	18	30.51
	<100	41	69.49
Gender	Mixed	46	77.97
	Males only	1	1.69
	Females only	12	20.34
Participant background	Existing mental health condition (Anxiety)	8	13.56
	Existing mental health condition (Depression)	2	3.39
	Enrolled as part of a course	9	15.25
	Referred to from psychology clinic	2	3.39
	Healthy (Physically/Mentally)	10	16.94
	Others (e.g. fatigue, poor sleep quality, stress, experience of negative life event, dance experience)	6	10.17
	Not reported	22	37.29
Intervention duration	4–6 weeks	14	23.73
	7–9 weeks	18	30.51
	10–12 weeks	17	28.81
	>12 weeks	10	16.95
Intervention frequency	1–2 times per week	23	38.98
	3–4 times per week	22	37.29
	5–7 times/week	8	13.56
	Other (gradual increase/self-directed)	2	3.39
	Not reported	4	6.78
Session duration	0–30 min	10	16.95
	30–60 min	18	30.51
	≥60 min	11	18.64
	Not reported	20	33.90
Type of physical activity*	Aerobic exercise (including dancing, walking, cheerleading and structured ball sports)	41	54.66
	Strength training	15	20
	Low-intensity/Low-impact exercise (including pilates, Tai Chi Quan, yoga, Ba Duan Jin, Kuok Sun Do)	19	25.33
	Education only	3	4
Intervention fidelity measures**	Reported	30	50.85
	Not reported	29	49.15
Measurement of physical activity	Reported	8	13.56
	Not reported	51	86.44
Intervention based on theory	Yes (list of theories used) 1. SAAFE principles (Lubans et al., 2017) 2. SMART goal setting Doran, 1981 3. Nies and Kershaw, (2002) model of PA and health outcomes 4. Motivational interviewing (Noonan & Moyers, 2009) 5. Social cognitive theory (Bandura, 2002) 6. Self-determination theory (Ryan & Deci, 2022)	5	8.47
	Not reported	54	91.53

*Note.* *Some interventions had more than one type of physical activity. ** Intervention fidelity was assessed according to whether authors reported attendance/adherence to the prescribed physical activity program.

**Table 2:** Description of studies included

Studies in meta-analysis						
Author/Year	Study design	Intervention and duration	Sample Size	Mental health outcome(s)	Behavioural theory	Quality
Abavisani *et al.*, (2019)	RCT	Pilates (8 weeks)	62 (31 PA; 31 control)	Anxiety	No	Weak
Akandere and Demir (2011)	RCT	Dance (12 weeks)	120 (60 PA; 60 control)	Depression	No	Weak
Akandere and Tekin (2008)	Non-RCT	Mixed (gymnastics, volleyball, athletics) (6 weeks)	143 (67 Yoga; 67 fitness)	Anxiety	No	Weak
Aşçı, (2002)	RCT	Step dance (10 weeks)	40 (20 PA, 20 control)	Anxiety	No	Weak
Balkin *et al.*, (2011)	Non-RCT	Unspecified aerobic exercise and strength training (6 weeks)	90 (21 anaerobic, 46 aerobic, 13 control)	Depression	No	Weak
Choi *et al.*, (2018)	RCT	Unspecified aerobic exercise and strength training (9 weeks)	63 (33 PA; 30 control)	Stress	Yes	Weak
de Vries *et al.*, (2017)	RCT	Low-intensity running (6 weeks)	97 (49 PA; 48 control)	Stress	No	Weak
Eather *et al.*, (2018)	RCT	High-intensity interval training (8 weeks)	53 (27 PA; 26 control)	Anxiety, stress	No	Weak
Erdoğan Yüce and Muz, (2020)	Non-RCT	Yoga (4 weeks)	89 (44 yoga; 45 control)	Anxiety, stress	No	Weak
Ghorbani et al. (2014)	RCT	Running and rope skipping (6 weeks)	30 (15 PA; 15 control)	Anxiety, depression	No	Weak
Herbert *et al*. (2020) Online only	RCT	Cardiovascular and muscular endurance exercises (6 weeks)	Online study: 61 (30 PA; 31 waitlist control)	Anxiety, depression	No	Strong
Hemat-Far et al. (2007)	RCT	Running (8 weeks)	20 (10 PA; 10 control)	Depression	No	Weak
Ji *et al.*, (2022)	RCT	Team sports, strength and aerobic exercises (7 weeks)	197 (66 team sports; 64 individual strength and aerobic training; 67 control)	Anxiety	No	Moderate
Kim, (2014)	RCT	Kuok Sun Do (4 weeks)	18 (7 PA; 11 control)	Anxiety, depression	No	Weak
Li and Li (2017)	RCT	Running and strength training (14 weeks)	37 (19 PA; 19 control)	Anxiety	No	Weak
Li *et al.*, (2015)	RCT	Baduanjin exercise (12 weeks)	206 (101 PA; 105 control)	Stress	No	Weak
Li *et al.*, (2022)	RCT	Resistance training (8 weeks)	27 (13 PA; 14 control)	Anxiety	No	Moderate
Ning (2020)	RCT	Unspecified aerobic exercise (20 weeks)	60 (30 PA; 30 control)	Anxiety, depression	No	Weak
Paolucci *et al.*, (2018)	RCT	High-intensity interval training and moderate-intensity training (6 weeks)	55 (18 HIIT; 19 mod-intensity training; 18 control)	Anxiety, depression, stress	No	Weak
Roth and Holmes, (2014)	RCT	Unspecified aerobic exercise (11 weeks)	55 (18 aerobic; 19 relaxation; 18 control)	Anxiety, depression, stress	No	Weak
Tomar *et al.*, (2023)	Non-RCT	Recreational team sports (12 weeks)	26 (16 PA; 10 control)	Depression	No	Moderate
Xiao *et al.*, (2021)	RCT	Basketball and baduanjin exercise (12 weeks)	96 (31 basketball, 31 baduanjin, 34 control)	Anxiety, stress	No	Weak
Yigiter and Hardee (2017)	RCT	Tennis and unspecified aerobic exercises (8 weeks)	60 (30 PA; 30 control)	Depression	No	Weak
Zhang *et al.*, (2023)	RCT	Tai Chi Quan (8 weeks)	18 (9 PA; 9 control)	Anxiety, depression	No	Moderate
Zheng *et al.*, (2015) Zheng *et al*. (2015) (Zheng et al., 2015)	RCT	Tai Chi Quan (12 weeks)	195 (92 PA; 103 control)	Stress	No	Weak

Studies in narrative synthesis						
Author/Year	Study design	Intervention	Sample size	Mental health outcome(s)	Behavioural theory	Quality
Ahmed et al. (2021)	Single-arm non-RCT	Pilates (6 weeks)	30 (no control)	Stress (*)	No	Weak
Alsaraireh and Aloush, (2017)	RCT	Unspecified aerobic exercise and strength training (10 weeks)	181 (90 exercise, 91 meditation)	Depression (*)	No	Moderate
Arbinaga *et al*. (2018)	RCT	Unspecified aerobic exercise (7 weeks)	141 (G1 no exercise, 13; G2, 15; G3, 17; G4, 21)	Wellbeing (+)	No	Moderate
BARĞI, (2022)	RCT	PA counselling (walking; 4 weeks)	31 (15 PA, 16 control)	Anxiety (+) Depression (/)	No	Moderate
Berger and Owen (1987)	Non-RCT	Swimming (14 weeks, study 1; 5 weeks, study 2)	100 (study 1: 52, study 2: 48—all were in swimming)	Anxiety (/)	No	Weak
Biber and Knoll, (2020)	Single-arm non-RCT	Strength training (12 weeks)	9 (no control)	Stress (*)	No	Weak
Caldwell *et al.*, (2011)	Non-RCT	Tai Chi Quan (15 weeks)	208 (76 taijiquan; 132 lectures/discussions control group)	Mindfulness (+) Stress (+)	No	Weak
Deng *et al.*, (2022)	Single-arm non-RCT	Cheerleading (16 weeks)	63 (no control)	Psychological symptoms (*)	No	Weak
Erdoğan Yüce and Muz, (2020)	Non-RCT	Yoga (4 weeks)	99 (44 yoga; 45 control)	Quality of life (*) Psychological health (*) Social relationships (*)	No	Weak
Forseth *et al.*, (2022)	Non-RCT	Yoga (8 weeks)	99 (44 PA; 45 control)	Depression (*) Stress (*)	No	Weak
Fukui *et al.*, (2021)	RCT	Strength training and balance exercises (8 weeks)	88 (39 PA; 49 control)	Psychological distress (+) Psychological wellbeing (+) Quality of life (/)	No	Weak
Gaskins *et al.*, (2020)	Single-arm non-RCT	Yoga (8 weeks)	19 (no control)	Stress (*)	No	Weak
Ghorbani *et al.*, (2014)	RCT	Running and rope skipping (6 weeks)	30 (15 PA; 15 control)	Social function/Loneliness (+)	No	Weak
Hamed *et al.*, (2021)	RCT	Running, cycling and strength training (8 weeks)	54 (27 PA; 27 control)	Anxiety (*) Depression (*) Stress (*)	No	Weak
Herbert *et al*. (2020) Lab Study	RCT	Cardiovascular and muscular endurance exercises (6 weeks)	Online study: 61 (30 PA; 31 waitlist control)	Anxiety (/) Depression (+) Coping strategy (/) Quality of life (/) Stress (+)	No	Strong
Huang (2021)	Single-arm non-RCT	Unspecified aerobic exercise (4 weeks)	240 (no control)	Psychological symptoms (*)	No	Weak
Johnston *et al.*, (2020)	Non-RCT	Running, dance, volleyball and soccer (12 weeks)	291 (138 team sports; 153 aerobic)	Anxiety (+) Depression (*) Stress (*)	No	Weak
Kim, (2014)	RCT	Yoga (12 weeks)	27 (12 PA; 15 control)	Stress (+)	No	Weak
Li and Liu, (2013)	Single-arm non-RCT	Running (16 weeks)	68 (no control)	Anxiety (*) Depression (*) Causes of stress (*)	No	Moderate
Li *et al.*, (2015)	RCT	Baduanjin exercise (12 weeks)	206 (101 PA; 105 control)	Quality of life (*) Psychological symptoms (*)	No	Weak
Mailey *et al*. (2010)	RCT	N/A—theory-focused intervention (12 weeks)	47 (24 PA; 23 control)	Anxiety (/) Depression (/)	Yes	Weak
Margulis *et al.*, (2021)	Non-RCT	Volleyball, soccer and strength training (12 weeks)	355 (140 team sports; 215 strength training)	Anxiety (/) Stress (/)	No	Weak
McCann and Holmes, (2006)	RCT	Unspecified aerobic exercise (10 weeks)	43 (15 aerobic; 14 placebos; 14 control)	Depression (+)	No	Weak
McFadden *et al.*, (2017)	Single-arm non-RCT	PA counselling—no specifics provided (8 weeks)	4 (no control)	Depression (*)	Yes	Weak
Murray *et al.*, (2022)	RCT	Unspecified aerobic training and resistance training (8 weeks)	74 (46 PA; 28 mindful)	Anxiety (/) Depression (/)	No	Strong
Sabourin *et al.*, (2016)	RCT	Running (14 weeks)	154 (125 CBT; 67 PA)	Anxiety (*) Depression (*) Distress (*)	No	Weak
Salehian *et al.*, (2021)	RCT	Qigong and resilience training (12 weeks)	45 (15 qigong; 15 resilience; 15 control)	Stress (*)	No	Weak
Schmalzl *et al.*, (2018)	RCT	Yoga (8 weeks)	40 (22 movement; 18 breath)	Stress (/)	No	Weak
Severtsen and Bruya, (2010)	RCT	Unspecified aerobic exercise (7 weeks)	10 (5 aerobic; 5 meditation)	Stress (*) Social readjustment/Loneliness (*)	No	Weak
Sharp and Caperchione, (2016)	RCT	Walking (12 weeks)	137 (72 PA; 65 control)	Psychological wellbeing (/) Mental health status (−)	Yes	Weak
Sun *et al.*, (2022)	RCT	Tai Chi Quan (16 weeks)	60 (30 PA; 30 control)	Psychological symptoms (*)	No	Weak
Terzioğlu *et al.*, (2022)	RCT	Mindfulness-based stretching (8 weeks)	60 (30 PA; 30 control)	Psychological wellbeing (+)	No	Weak
Tomar *et al.*, (2023)	Non-RCT	Recreational team sports (12 weeks)	26 (16 PA; 10 control)	Psychological wellbeing (*)	No	Moderate
Tong *et al.*, (2020)	RCT	Yoga, running and strength training (12 weeks)	143 (67 Yoga; 67 fitness)	Stress (+) Mindfulness (+)	No	Weak
Tucker and Maxwell, (2011)	Non-RCT	Strength training (15 weeks)	152 (60 PA; 92 control)	Wellbeing (+)	No	Moderate
Wang *et al.*, (2023)	RCT	Aerobic and resistance exercises (8 weeks)	49 (24 HIIT, 25 aerobic)	Psychological symptoms (/)	No	Moderate
Yazici *et al.*, (2016)	Single-arm non-RCT	Tennis (13 weeks)	76 (no control)	Anxiety (*) Depression (*) Psychological symptoms (*)	No	Moderate
Zhang and Luo, (2020)	Non-RCT	Water sports (8 weeks)	800 (400 PA; 400 control)	Psychological symptoms (*)	No	Weak
Zheng *et al.*, (2015)	RCT	Tai Chi Quan (12 weeks)	195 (92 PA; 103 control)	Psychological symptoms (/) Quality of life (/) Stress (/)	No	Weak
Zheng *et al.*, (2021)	RCT	Dance (8 weeks)	181 (90 PA; 91 control)	Anxiety (*) Loneliness (*) Psychological stress (*)	No	Weak

*Note*: Specific behavioural change theories are mentioned in text. Some studies do not report the specific aerobic/anaerobic exercise. Weight training and strength training were used interchangeably and had been standardized as strength training here. Legend:  +  (statistically positive effect); − (statistically negative effect); / (statistically non-significant effect); * (insufficient evidence).

### Meta-analysis findings

**Fig. 2:**
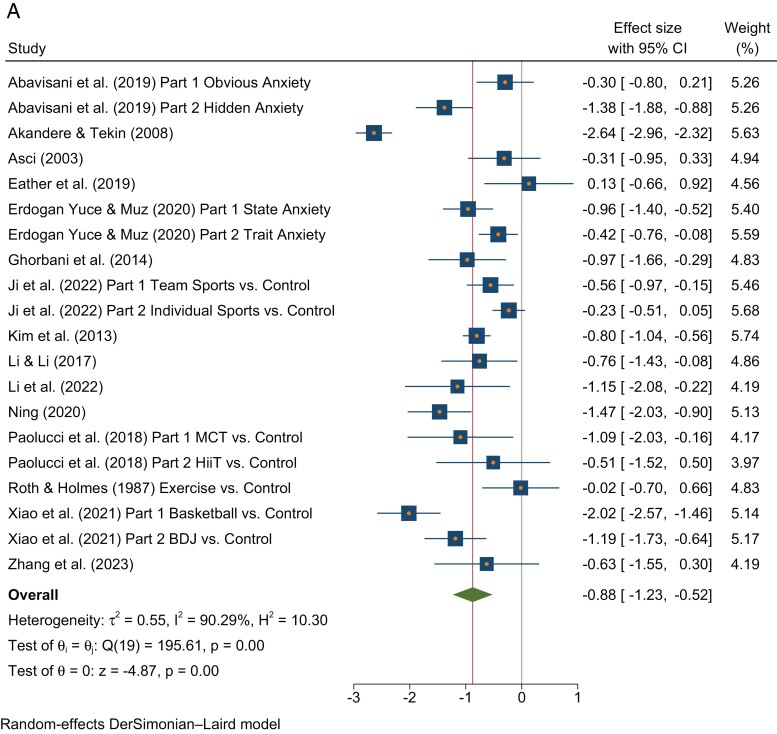

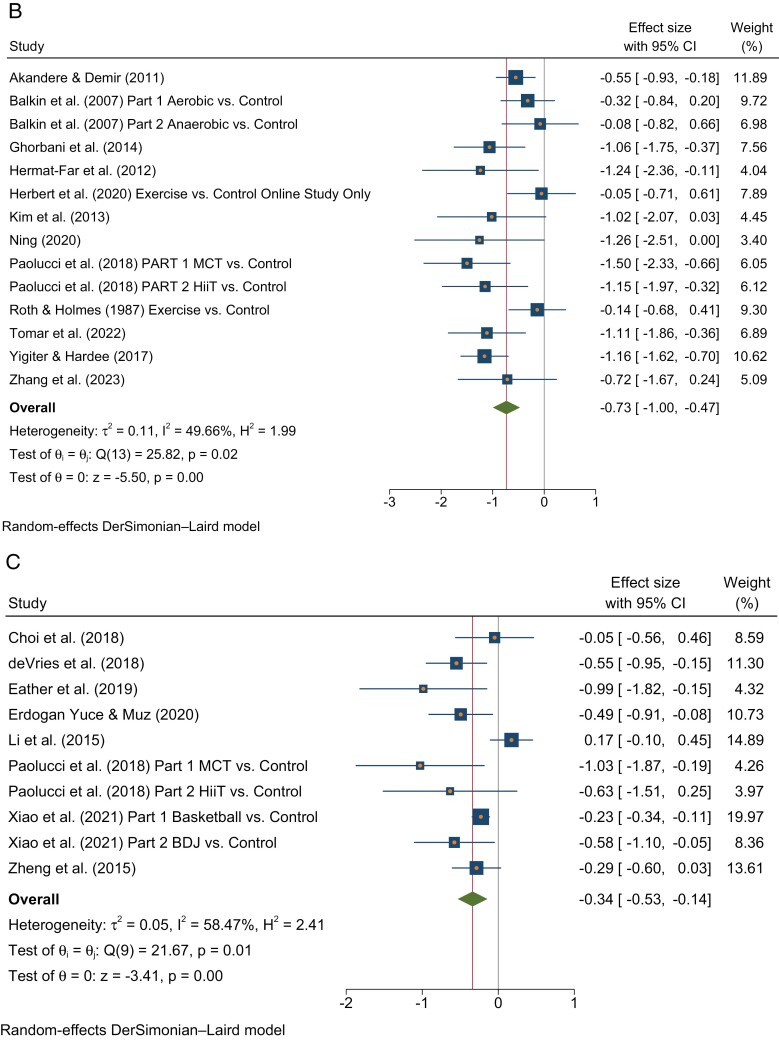
(a) Forest Plot for Anxiety. (b) Forest Plot for Depression. (c) Forest Plot for Stress after removing Kim, (2014).

The pooled effects indicated that the PA interventions showed moderate to large effects (Cohen, 2013) in reducing poor mental health outcomes in undergraduate students in terms of anxiety (*N* = 20, pooled standardized mean difference (SMD) = −0.88, 95% CI [−1.23, −0.52]), depression (*N* = 14, pooled SMD = −0.73, 95% CI [−1.00, −0.47]) and stress (*N* = 11, pooled SMD = −0.61, 95% CI [−0.94, −0.28]). Heterogeneity was moderately high and statistically significant for anxiety (*I*^*2*^ = 90.29%; *Q*(19) = 195.61, *p* < 0.001), depression (*I*^*2*^ = 49.66%; *Q*(13) = 25.82, *p* = 0.02) and stress (*I*^*2*^ = 86.97%; *Q*(10) = 76.74, *p* < 0.001). A list of the studies included in the meta-analysis can be found in Table 2. The variable findings underpinning heterogeneity were mostly in the form of almost all studies pointing towards benefit but to differing degrees (as can be seen in the forest plots for anxiety, depression and stress presented in Figure 2), rather than a mix of studies showing benefits and detrimental impacts. Begg’s test for publication bias was not statistically significant for anxiety (*z* = −0.32, *p* = 0.795) and depression (*z* = −0.88, *p* = 0.443), but was significant for stress (*z* = −2.18, *p* = 0.043). The trimmed and filled estimate for stress was (−0.22, 95% CI [−0.31, −0.14]).

The leave-one-out sensitivity analyses indicated the findings for anxiety and depression were robust to study inclusion, with pooled effects remaining moderately sized and statistically significant regardless of which studies were included, with results ranging from −0.77, 95% CI [−1.00, −0.54] to −0.92, 95% CI [−1.28, −0.57] for anxiety and −0.68, 95% CI [−0.94, −0.41] to −0.79, 95% CI [−1.05, −0.43] for depression, still with significant heterogeneity. Conclusions regarding stress were affected by study inclusion. The omission of most studies did not affect the pooled effects, which ranged from −0.58, 95% CI [−0.92, −0.24] to −0.72, 95% CI [−1.08, −0.36]. However, omitting the findings of Kim, (2014), led to a much smaller effect, −0.34, 95% CI [−0.53, −0.14], which was still heterogeneous (*I*^*2*^ = 58.47%; *Q*(9) = 21.67, *p* = 0.01) but no longer showed small study effects (*z* = −1.97, *p* = 0.074). Pooled effects in the trimmed and filled analyses were −0.26, 95% CI [−0.45, −0.07] after omitting Kim (2014).

### Narrative synthesis results

The narrative synthesis included 40 studies that reported on 12 mental health outcomes. Descriptive data for studies included in the narrative synthesis are summarized in Table 2 and a synthesis of findings per outcome is available in Supplementary Material A, Table S4. Anxiety, depression and stress were the most assessed mental health outcomes in studies included in the narrative synthesis, which is consistent with the outcomes included in the meta-analyses. Overall, across outcomes, the synthesis of findings showed 22% positive effects, 26% insignificant effects and only about 1% negative effects. However, 51% (37 out of 73) instances were unable to be coded due to a lack of appropriate statistical analyses (e.g. use of independent samples *t*-tests instead of ANOVAs, even where comparison groups were available), a lack of reporting of group by time interactions, or studies using single-arm trials with no comparison group. When looking at specific outcomes, psychological wellbeing had the highest proportion of statistically significant positive effects (4 out of 6, or 67%).

## DISCUSSION

This systematic review and meta-analysis examined the effectiveness of PA interventions on undergraduate students’ mental health outcomes. Meta-analyses showed that PA interventions had a significant, moderate effect on reducing anxiety and depression and an uncertain (moderate or smaller), but significant, effect on reducing stress. In contrast, the narrative synthesis found mixed findings for the effectiveness of PA interventions in improving mental health outcomes in undergraduate students, with only 22% significant positive effects across 12 different mental health outcomes. Across both the meta-analyses and narrative synthesis, we observed significant variability in the parameters of prescribed PA interventions (e.g. frequency, intensity, duration and type). About 80% of included papers demonstrated low quality and the certainty of evidence (GRADE) was very low for the three outcomes included in meta-analyses. Findings should be interpreted with this information in mind.

The findings from our meta-analyses are generally consistent with existing literature, which suggests that engaging in PA can be beneficial to university students’ mental health (e.g. Hrafnkelsdottir *et al*., 2018; Román-Mata *et al*., 2020; Reyes-Molina *et al*., 2022; Ruiz-Hernández *et al*., 2022). Our findings are also consistent with those of a scoping review focusing on the impact of PA on the mental health of young people aged 12–26 years (Pascoe *et al*., 2020), with both studies indicating positive improvements for anxiety and depression but our results also supporting improvements in symptoms of stress. Specifically focusing on university students, our findings complement those of a previous systematic review and meta-analysis that reported that PA interventions increased moderate intensity PA in university students (Plotnikoff *et al*., 2015), by also showing that PA interventions can benefit mental health outcomes. Recent data suggest that while participation rates are still low, university students are amongst the most physically active, but still experience the highest onsets of mental health disorders compared to other age groups (ABS, 2023). One possible explanation could be the gap between university students’ understanding of PA as a mental health strategy (Dingle *et al*., 2022). Future research could address the discrepancy between students’ knowledge of PA as a coping strategy to mitigate the effects of poor mental health and the actual utilization of PA.

Given the high heterogeneity in our meta-analyses and inconclusive findings from the narrative synthesis, it may be reasonable to conclude that PA interventions do not have a single fixed impact on mental health outcomes (Higgins *et al*., 2002; Imrey, 2020). The high heterogeneity reflects the high level of variability in the types of PA interventions included in our review in terms of type (e.g. aerobic exercises, low-intensity exercises like yoga and pilates and strength training), intervention duration (e.g. 4–16 weeks), session frequency, session duration and inclusion criteria for participants. Thus, the generalisability of our findings to other PA interventions may be limited, particularly if they are different to the studies included in this review and shown to be effective (Higgins *et al*., 2002; Imrey, 2020). Our results are consistent with existing systematic reviews indicating highly heterogeneous PA interventions in university students (Dogra *et al*., 2018; Pascoe *et al*., 2020; Wunsch *et al*., 2021). However, the positive effects found for mental health outcomes despite the variability of PA interventions might suggest that university students will reap mental health benefits regardless of the type and dose of PA intervention they are exposed to. Our results provide further empirical evidence to recommend PA guidelines, which stipulate that any PA is going to be beneficial not only physically but also psychologically (WHO, 2023). There is some evidence that intensities may vary the effect of PA on mental health (Pascoe *et al*., 2020), but this was not analysed in the current study. Future studies should at least simultaneously focus on the behaviour change aspects (e.g. including directly measuring PA) of their intervention, as well as the PA prescription parameters (e.g. such as focusing on sub-group analyses of intensities), to promote habit formation, uptake and maintenance. It is also worth considering that rather than the direct potential benefit of PA alone, PA performed with others could also provide students with opportunities to connect socially and form better sources of support, which may also bring benefits to their psychological wellbeing via increasing belonging to their university and community (Stevens *et al*., 2017; Dingle *et al*., 2021). Future studies could potentially include control-comparison methods to identify the extent to which social aspects in PA interventions improve mental health outcomes.

Understanding the development of PA interventions and the fidelity to the protocol is critical in interpreting findings in PA research. PA is a health behaviour, and conceptual or theoretical underpinnings, such as behavioural change frameworks, can provide evidence-informed design of interventions by intentionally targeting key determinants of change (e.g. psychological, social and environmental factors) (Michie *et al*., 2011). Furthermore, information about intervention fidelity, such as typical attendance, or adherence to the prescribed dose of PA, and measurement of PA as an outcome measure can facilitate a better understanding of the effectiveness (or lack thereof) of interventions (Love *et al*., 2019). In our review, only five of 59 studies reported using a behavioural change theory in the design of their PA intervention (Mailey *et al*., 2010; Sharp and Caperchione, 2016; McFadden *et al.*, 2017; Choi *et al.*, 2018; Eather *et al.*, 2018) and only ~50% of studies reported some information regarding intervention fidelity. This means that in 50% of the studies, whether participants received the prescribed dose of PA is unknown. Considering that PA interventions are in essence, behaviour-focused interventions (Hagger, 2019), our findings highlight a greater need for future PA interventions to incorporate, and report, behavioural change frameworks and report intervention fidelity.

Considering the increasing rates of poor mental health in undergraduate students (Lipson *et al*., 2022) and the positive effect of PA on mental health identified in the current meta-analysis, PA interventions have the potential to be an effective strategy to prevent and manage university students’ mental health (e.g. Carballo-Fazanes *et al*., 2020; deJonge *et al*., 2020). Implementing PA as a prevention strategy could help to ease the existing long waitlists students experience when attempting to assess professional mental health services (e.g. Broglia *et al*., 2018; Dingle *et al*., 2021). Encouraging students to engage in early intervention strategies may be beneficial long-term, as it may protect students from ever reaching diagnostic thresholds for mental ill health (Duffy *et al*., 2020). Prompt intervention could not only mitigate the potential long-term impacts of poor mental health on academic performance (Babaeer *et al*., 2020), relationships and overall wellbeing (Duffy *et al*., 2020), but it could also set students up with a higher chance of experiencing successful recovery from mental ill health (Colizzi *et al*., 2020).

The current systematic review is the first to examine the effects of PA interventions on improving mental health outcomes in undergraduate students. A strength of this review involves the use of a meta-analysis for selected outcomes; meta-analyses are ranked highly when accessing levels of evidence (National Health and Medical Research Council, 2009). The review employed a thorough search strategy, utilizing a broad range of databases and screening over 13,000 potential studies, increasing confidence that the search strategy has captured the available evidence related to the effectiveness of PA interventions for improving undergraduate students’ mental health. Another strength was that our strict eligibility criteria excluded studies that could have contributed additional confounding variables (e.g. we excluded studies that targeted more than one health behaviour in a single intervention arm and studies that had samples with both undergraduate and postgraduate students), which allowed us to isolate the effect of PA interventions on our target population. Lastly, our review joins a small but growing body of systematic reviews examining the use of behaviour change theories in intervention design and fidelity for PA interventions targeting mental health outcomes in undergraduate students (Kahn *et al*., 2002; Maselli *et al*., 2018; Wibowo *et al*., 2020).

Several limitations must also be considered. All eligible studies for the meta-analysis were not able to be included; nine studies were excluded as relevant data were not able to be retrieved from authorship teams. Another 51% of studies in the narrative synthesis could not be coded because of insufficient evidence. While the meta-analyses provide the first quantitative synthesis on this topic, it must also be acknowledged that the findings should be interpreted in the context of the poor overall study quality and high heterogeneity of included studies. A larger sample for the meta-analysis may have yielded different results, as well as made it possible to explore additional questions (such as sub-analyses by intervention type or by group vs. individual PA). Studies not published in English were not included, potentially excluding relevant research. Publication bias, where studies with little to no effect tend to remain unpublished (Franco *et al*., 2014), was considered by testing for small-study effects, and providing corrected estimates where needed, but these approaches have limitations, and it is still possible that the inclusion of unpublished studies could have led to different results. This study only included undergraduate students, so the findings can’t be generalized to postgraduate students, who may have unique needs and experiences at university. Lastly, the studies included were at high risk of bias, which limited the confidence of our conclusions.

## CONCLUSION

With increasing rates of poor mental health among undergraduate students at university, PA has the potential to reduce overall incidence. The findings from the current systematic review and meta-analyses suggest that PA interventions of at least 4 weeks duration are moderately effective at reducing symptoms of mental ill health (depression, anxiety and stress) in undergraduate students. However, these results should be interpreted with caution due to the high risk of bias. Future research should focus on improving the methodological quality of studies, developing interventions that are theoretically informed by evidence-based behavioural change theories and techniques and improving reporting of intervention fidelity.

## Supplementary Material

daae054_suppl_Supplementary

## Data Availability

Data related to this study such as the search strategy, raw data from meta-analyses, descriptive data from meta-analyses and narrative synthesis, forest plots not in the manuscript, review references, figures and tables are attached as Supplementary material in the appendices. The STATA code used for meta-analyses can be provided upon request.
