# Supplementary material for: Effectiveness of physical activity interventions on undergraduate students’ mental health: systematic review and meta-analysis
Source: Health Promot Int. 2024 Jun 25;39(3):daae054. doi: 10.1093/heapro/daae054 (PMC11196957; doi:10.1093/heapro/daae054)
Supplement: daae054_suppl_Supplementary [file daae054_suppl_supplementary.zip › Huang_Appendix_A_Search_Strategy.docx]

**Appendix A: Search Strategy**

PubMed

(("student*"[Title/Abstract] OR "Students"[MeSH Terms]) AND ("undergrad*"[Title/Abstract] OR "college*"[Title/Abstract] OR "higher education"[Title/Abstract] OR "tertiary*"[Title/Abstract] OR "universit*"[Title/Abstract])) OR "Universities"[MeSH Terms]

*PA Subject Heading (MeSH) not included because PubMed translate PA=exercise*

"circuit training"[Title/Abstract] OR "physical exerci*"[Title/Abstract] OR "physical endurance"[Title/Abstract] OR "physical train*"[Title/Abstract] OR "Motor Activity"[MeSH Terms] OR "exercise"[MeSH Terms] OR "exerci*"[Title/Abstract] OR "sport*"[Title/Abstract] OR "physical activi*"[Title/Abstract] OR "physical fitness"[Title/Abstract] OR "resistance training"[Title/Abstract] OR "aerobic*"[Title/Abstract] OR "strength training"[Title/Abstract]

("mental*"[Title/Abstract] OR anxiety[Title/Abstract] OR depression[Title/Abstract] OR "wellbeing*"[Title/Abstract] OR "stress*"[Title/Abstract] OR "coping*"[Title/Abstract] OR "loneli*"[Title/Abstract] OR "social isolation"[Title/Abstract] OR "distress*"[Title/Abstract] OR ("Mental Health"[MeSH Terms] OR "Mental Disorders"[MeSH Terms]))

Embase

(student*:ti,ab OR 'students'/exp) AND (undergrad*:ti,ab OR college*:ti,ab OR 'higher education':ti,ab OR tertiary*:ti,ab OR universit*:ti,ab) OR 'universities'/exp

- Proximity search: (student* NEAR/5 (undergrad* OR college* OR 'higher education' OR tertiary* OR universit*)):ab,ti

'circuit training':ti,ab OR 'physical exerci*':ti,ab OR 'physical endurance':ti,ab OR 'physical train*':ti,ab OR 'motor activity'/exp OR 'exercise'/exp OR exerci*:ti,ab OR sport*:ti,ab OR 'physical activi*':ti,ab OR 'physical fitness':ti,ab OR 'resistance training':ti,ab OR aerobic*:ti,ab OR 'strength training':ti,ab

(mental*:ti,ab OR anxiety:ti,ab OR depression:ti,ab OR wellbeing*:ti,ab OR stress*:ti,ab OR coping*:ti,ab OR loneli*:ti,ab OR 'social isolation':ti,ab OR distress*:ti,ab OR ('Mental Health'/exp OR 'Mental Disorders'/exp))

Cinahl
(((TI student* OR AB student*) OR (MH Students+)) AND ((TI undergrad* OR AB undergrad*) OR (TI college* OR AB college*) OR (TI "higher education" OR AB "higher education") OR (TI tertiary* OR AB tertiary*) OR (TI universit* OR AB universit*))) OR (MH Universities+)

- Proximity: TI (student* N5 (undergrad* OR college* OR "'higher education'" OR tertiary* OR universit* )) OR AB (student* N5 (undergrad* OR college* OR "'higher education'" OR tertiary* OR universit* ))

(MH "Motor Activity+") OR (MH exercise+) OR (TI exerci* OR AB exerci*) OR (TI sport* OR AB sport*) OR (TI "physical activi*" OR AB "physical activi*") OR (TI "physical fitness" OR AB "physical fitness") OR (TI "resistance training" OR AB "resistance training") OR (TI aerobic* OR AB aerobic*) OR (TI "strength training" OR AB "strength training")

((TI mental* OR AB mental*) OR (TI anxiety OR AB anxiety) OR (TI depression OR AB depression) OR (TI wellbeing* OR AB wellbeing*) OR (TI stress* OR AB stress*) OR (TI coping* OR AB coping*) OR (TI loneli* OR AB loneli*) OR (TI "social isolation" OR AB "social isolation") OR (TI distress* OR AB distress*) OR ((MH "Mental Health+") OR (MH "Mental Disorders+")))

SPORTDiscus

(((TI student* OR AB student*) OR (MH Students+)) AND ((TI undergrad* OR AB undergrad*) OR (TI college* OR AB college*) OR (TI "higher education" OR AB "higher education") OR (TI tertiary* OR AB tertiary*) OR (TI universit* OR AB universit*))) OR (MH Universities+)

- Proximity: (((TI student* OR AB student*) OR (MH Students+)) AND ((TI undergrad* OR AB undergrad*) OR (TI college* OR college*) OR (TI "higher education" OR AB "higher education") OR (TI tertiary* OR AB tertiary*) OR (TI universit* OR AB universit*))) OR (MH Universities+)

(MH "Motor Activity+") OR (MH exercise+) OR (TI exerci* OR AB exerci*) OR (TI sport* OR AB sport*) OR (TI "physical activi*" OR AB "physical activi*") OR (TI "physical fitness" OR AB "physical fitness") OR (TI "resistance training" OR AB "resistance training") OR (TI aerobic* OR AB aerobic*) OR (TI "strength training" OR AB "strength training")

((TI mental* OR AB mental*) OR (TI anxiety OR AB anxiety) OR (TI depression OR AB depression) OR (TI wellbeing* OR AB wellbeing*) OR (TI stress* OR AB stress*) OR (TI coping* OR AB coping*) OR (TI loneli* OR AB loneli*) OR (TI "social isolation" OR AB "social isolation") OR (TI distress* OR AB distress*) OR ((MH "Mental Health+") OR (MH "Mental Disorders+")))

SCOPUS

((TITLE-ABS-KEY({circuit training}) OR TITLE-ABS-KEY("physical exerci*") OR TITLE-ABS-KEY({physical endurance}) OR TITLE-ABS-KEY("physical train*") OR (TITLE-ABS-KEY({Motor Activity}) OR TITLE-ABS-KEY(exerci*) OR TITLE-ABS-KEY(sport*) OR TITLE-ABS-KEY("physical activi*") OR TITLE-ABS-KEY({physical fitness}) OR TITLE-ABS-KEY({resistance training}) OR TITLE-ABS-KEY(aerobic*) OR TITLE-ABS-KEY({strength training})))) AND ((((TITLE-ABS-KEY(students)) AND (TITLE-ABS-KEY(undergrad*) OR TITLE-ABS-KEY(college*) OR TITLE-ABS-KEY({higher education}) OR TITLE-ABS-KEY(tertiary) OR TITLE-ABS-KEY(universit*))) )) AND ((TITLE-ABS-KEY(mental*) OR TITLE-ABS-KEY(anxiety) OR TITLE-ABS-KEY(depression) OR TITLE-ABS-KEY(wellbeing*) OR TITLE-ABS-KEY(stress*) OR TITLE-ABS-KEY(coping*) OR TITLE-ABS-KEY(loneli*) OR TITLE-ABS-KEY({social isolation}) OR TITLE-ABS-KEY(distress*)))

Web of Science

(((TI=student* OR AB=student*) OR ALL=Students) AND ((TI=undergrad* OR AB=undergrad*) OR (TI=college* OR AB=college*) OR (TI="higher education" OR AB="higher education") OR (TI=tertiary* OR AB=tertiary*) OR (TI=universit* OR AB=universit*)))

(TI="circuit training" OR AB="circuit training") OR (TI="physical exerci*" OR AB="physical exerci*") OR (TI="physical endurance" OR AB="physical endurance") OR (TI="physical train*" OR AB="physical train*") OR (TI="Motor Activity" OR AB=”Motor Activity”) OR (TI=exerci* OR AB=exerci*) OR (TI=sport* OR AB=sport*) OR (TI="physical activi*" OR AB="physical activi*") OR (TI="physical fitness" OR AB="physical fitness") OR (TI="resistance training" OR AB="resistance training") OR (TI=aerobic* OR AB=aerobic*) OR (TI="strength training" OR AB="strength training")

((TI=mental* OR AB=mental*) OR (TI=anxiety OR AB=anxiety) OR (TI=depression OR AB=depression) OR (TI=wellbeing* OR AB=wellbeing*) OR (TI=stress* OR AB=stress*) OR (TI=coping* OR AB=coping*) OR (TI=loneli* OR AB=loneli*) OR (TI="social isolation" OR AB="social isolation") OR (TI=distress* OR AB=distress*))

PSYCINFO

((title: (Student*)) OR (abstract: (Student*))) OR ((IndexTermsFilt: ("Students")))

AND

((title: ("undergrad*") OR title: ("college*") OR title: ("higher education") OR title: ("tertiary*") OR title: ("universit*")) OR (abstract: ("undergrad*") OR abstract: ("college*") OR abstract: ("higher education") OR abstract: ("tertiary*") OR abstract: ("universit*"))) OR ((IndexTermsFilt: ("Colleges")))

AND

((**title**: ("mental*")) *OR* (**title**: (anxiety)) *OR* (**title**: (depression)) *OR* (**title**: ("wellbeing*")) *OR* (**title**: ("stress*")) *OR* (**title**: ("coping*")) *OR* (**title**: ("loneli*")) *OR* (**title**: ("social isolation")) *OR* (**title**: ("distress*")) )

OR

((**abstract**: ("mental*")) *OR* (**abstract**: (anxiety)) *OR* (**abstract**: (depression)) *OR* (**abstract**: ("wellbeing*")) *OR* (**abstract**: ("stress*")) *OR* (**abstract**: ("coping*")) *OR* (**abstract**: ("loneli*")) *OR* (**abstract**: ("social isolation")) *OR* (**abstract**: ("distress*")) )

AND

((**title**: ("circuit training")) *OR* (**title**: ("physical exerci*")) *OR* (**title**: ("physical endurance")) *OR* (**title**: ("physical train*")) *OR* (**title**: ("exerci*")) *OR* (**title**: (sport*)) *OR* (**title**: ("physical fitness")) *OR* (**title**: ("resistance training")) *OR* (**title**: ("aerobic*")) *OR* (**title**: ("strength training")) )

OR

((**abstract**: ("circuit training")) *OR* (**abstract**: ("physical exerci*")) *OR* (**abstract**: ("physical endurance")) *OR* (**abstract**: ("physical train*")) *OR* (**abstract**: ("exerci*")) *OR* (**abstract**: (sport*)) *OR* (**abstract**: ("physical fitness")) *OR* (**abstract**: ("resistance training")) *OR* (**abstract**: ("aerobic*")) *OR* (**abstract**: ("strength training")) )
