# Supplementary material for: Effectiveness of physical activity interventions on undergraduate students’ mental health: systematic review and meta-analysis
Source: Health Promot Int. 2024 Jun 25;39(3):daae054. doi: 10.1093/heapro/daae054 (PMC11196957; doi:10.1093/heapro/daae054)
Supplement: daae054_suppl_Supplementary [file daae054_suppl_supplementary.zip › Huang_Supplementary_A_Tables_Figures_NotinManuscript.docx]

| **Table 3.**  Certainty of Evidence (GRADE) | | | | | | |
| --- | --- | --- | --- | --- | --- | --- |
| Outcomes | No. of Studies | Limitation | Inconsistency | Indirectness | Imprecision | Overall GRADE |
| Anxiety | 20 (14 RCTs) | Serious Limitations | Serious Inconsistency | Serious Indirectness | Serious Imprecision | Very Low |
| Depression | 14 (9 RCTs) | Serious Limitations | Moderate Inconsistency | Serious Indirectness | Serious Imprecision | Very Low |
| Stress | 10 (8 RCTs) | Serious Limitations | Moderate Inconsistency | Serious Indirectness | Serious Imprecision | Very Low |
| *Note.* Population: Undergraduate students; Intervention: PA; Study Designs included: Trials that included one intervention group compared with a control group. GRADE levels of evidence: Very Low Certainty indicates that we have very little confidence in the effect estimate. | | | | | | |

**Supplementary Material A: Tables not in Manuscript**

| **Table 4.** Summary of Narrative Synthesis by Outcome | | | | | | |
| --- | --- | --- | --- | --- | --- | --- |
| **No.** | **Outcome** | **+** | **-** | **/** | ***** | **Total per Outcome** |
| 1 | Anxiety | 2 |  | 5 | 5 | 12 |
| 2 | Causes of Stress |  |  |  | 1 | 1 |
| 3 | Coping Strategy |  |  | 1 |  | 1 |
| 4 | Depression | 2 |  | 3 | 8 | 13 |
| 5 | Loneliness (Social Relationships) | 1 |  |  | 3 | 4 |
| 6 | Mindfulness | 2 |  |  |  | 2 |
| 7 | Psychological Distress | 1 |  |  | 1 | 2 |
| 8 | Psychological Health/Mental Health Status |  | 1 |  | 1 | 2 |
| 9 | Psychological Symptoms |  |  | 2 | 6 | 8 |
| 10 | Psychological Wellbeing | 4 |  | 1 | 1 | 6 |
| 11 | Quality of Life |  |  | 4 | 2 | 6 |
| 12 | Stress | 4 |  | 3 | 9 | 16 |
|  | **Total per Result** | 16 (21.92%) | 1 (1.37%) | 19 (26.03%) | 37 (50.68%) | 73 |

**Supplementary Material B: Figures not in Manuscript**

**Figure. 3.** MERST Breakdown of each component of quality appraisal. *Needs to be in colour*


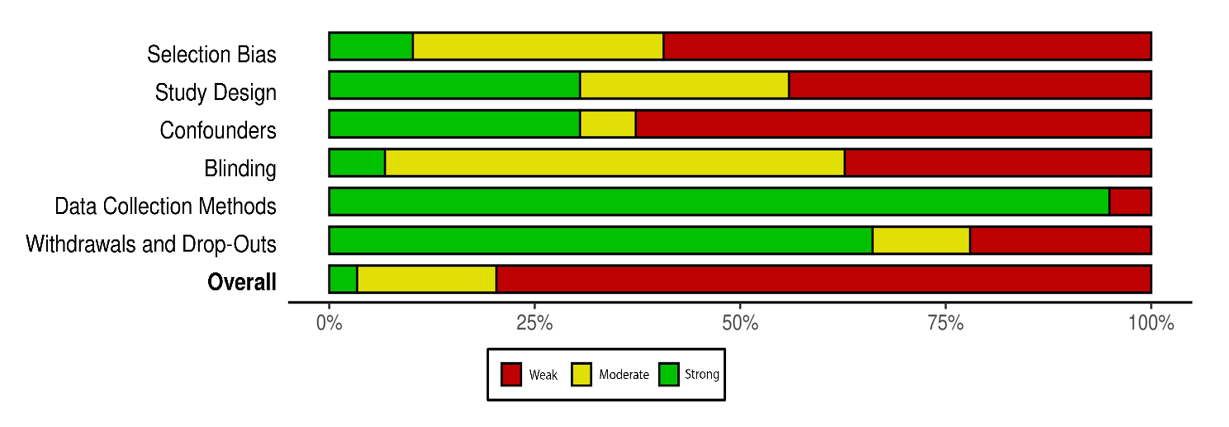


**Supplementary Material C: Forest Plots not in Manuscript**

**
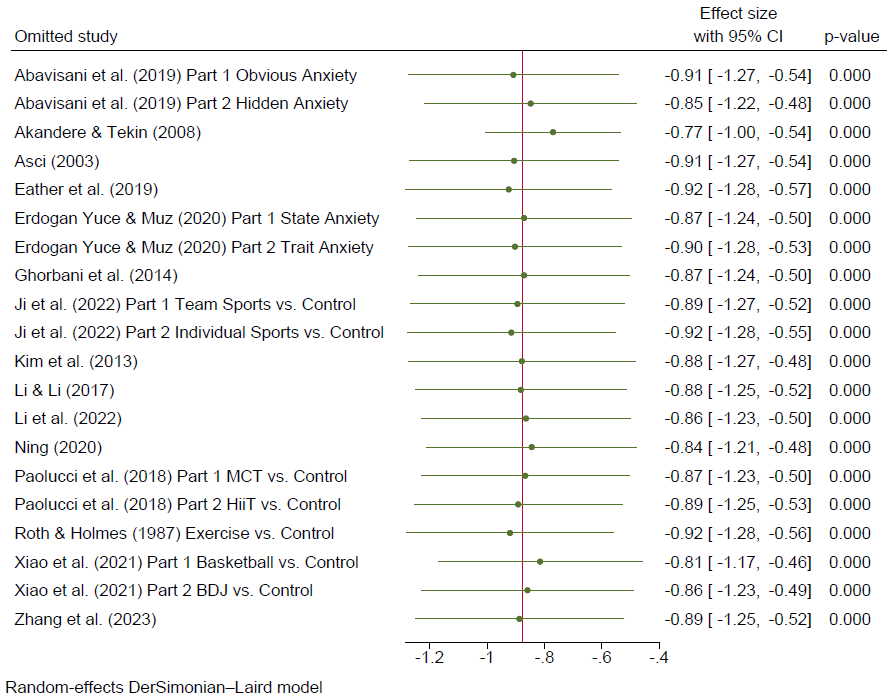
Anxiety Leave-One-Out Forest Plot**

**
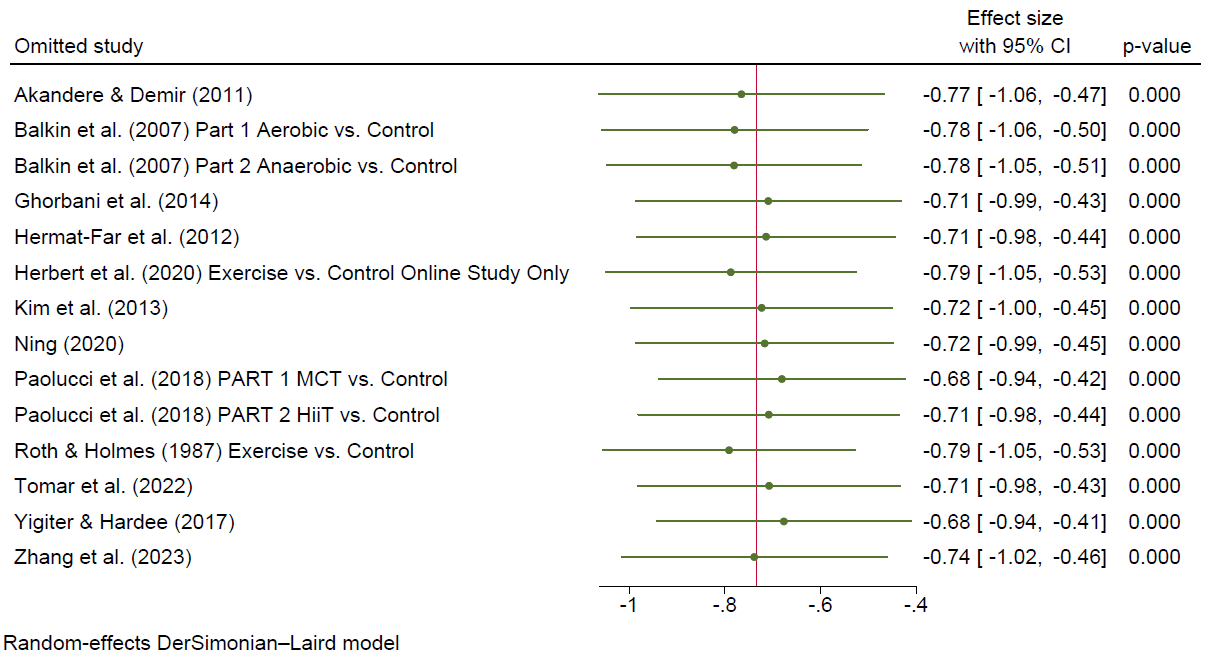
Depression Leave-One-Out Forest Plot**

**
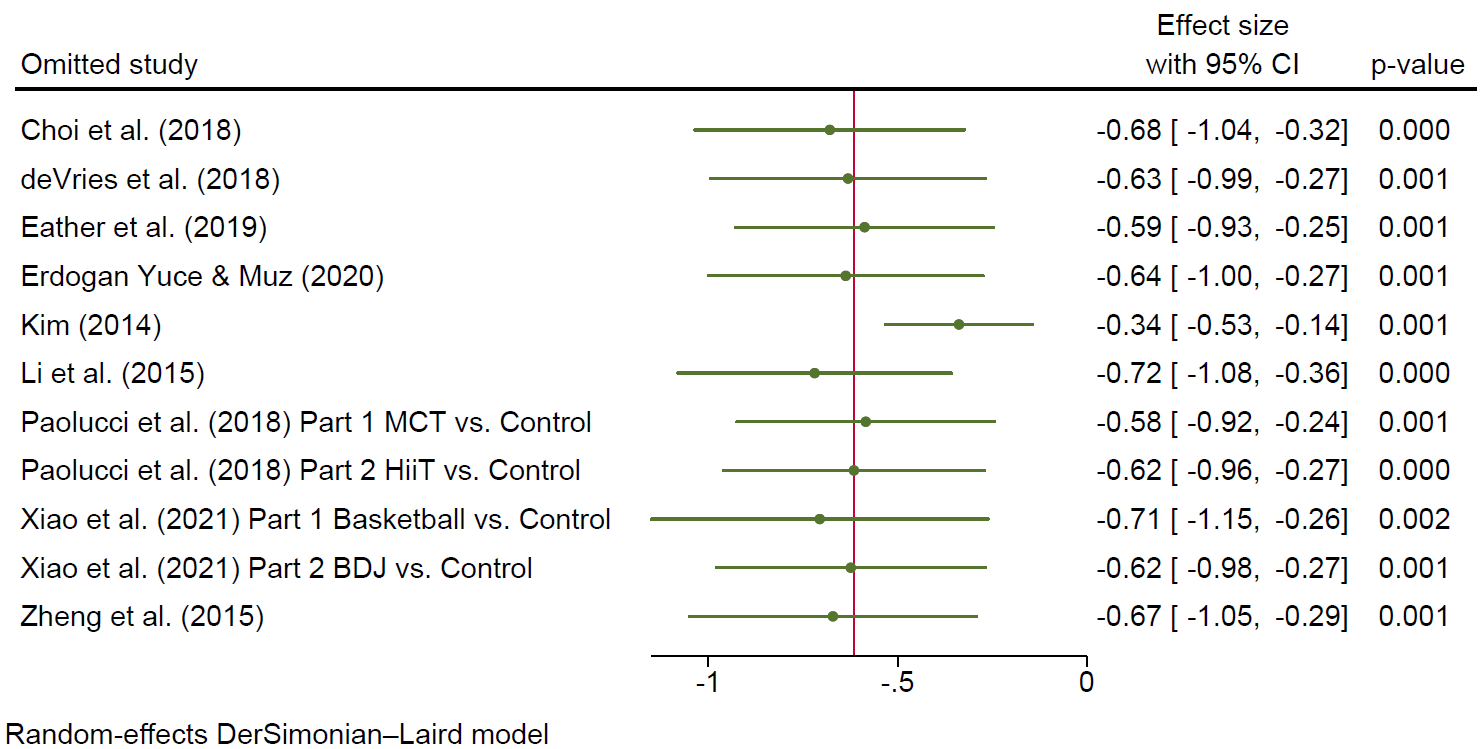
Stress Leave-One-Out Forest Plot**

**Stress Random Effects Meta-analysis (with Kim, 2014)**

**
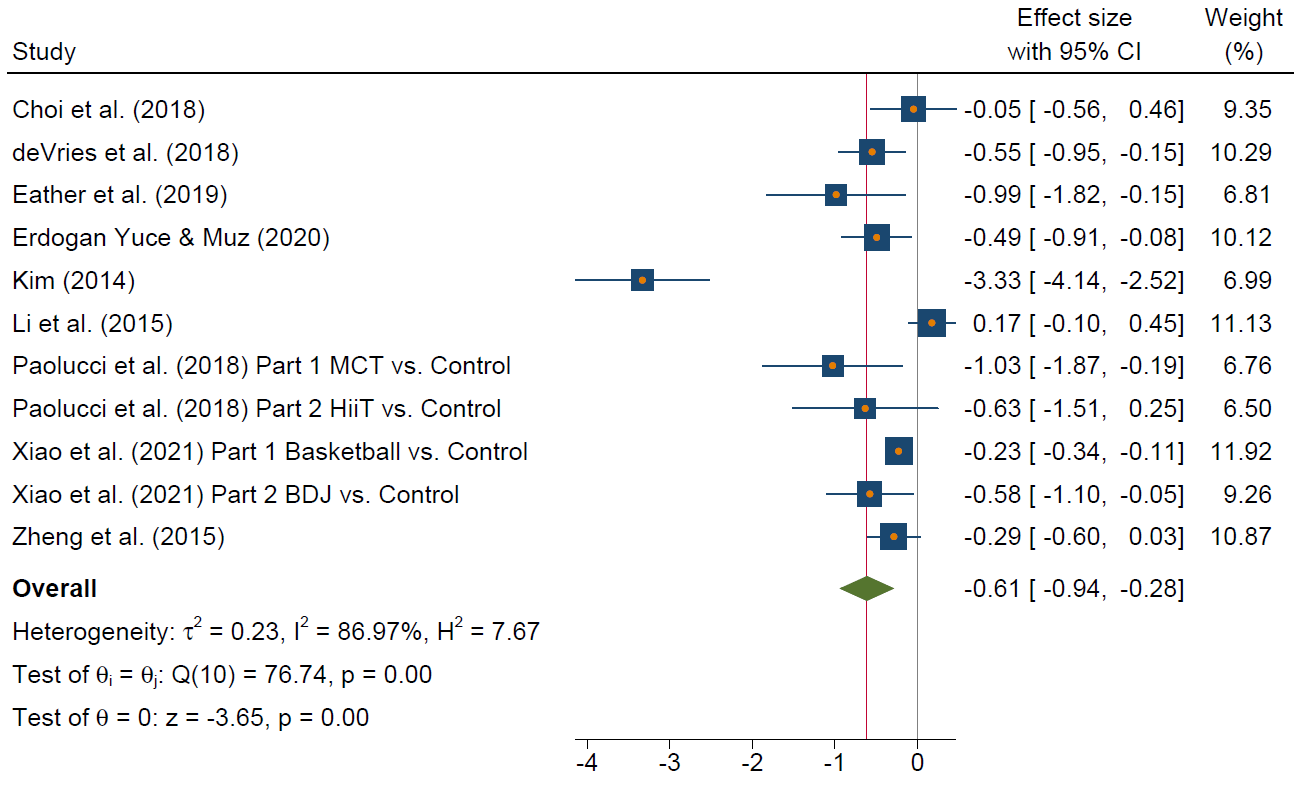
**
