# Supplementary material for: Effectiveness of physical activity interventions on undergraduate students’ mental health: systematic review and meta-analysis
Source: Health Promot Int. 2024 Jun 25;39(3):daae054. doi: 10.1093/heapro/daae054 (PMC11196957; doi:10.1093/heapro/daae054)
Supplement: daae054_suppl_Supplementary [file daae054_suppl_supplementary.zip › Huang_Supplementary_D_Meta_Raw_Data.pdf]

StudyNumber_Author	Outcome	IxEffectStd_M	IxEffectUnitsStd_SE	Intervention Group Effect Units Mean	Intervention Group Effect Units Standard Error	MeanUnits_Pre_Intervention Group	SDUnits_Pre_Intervention Group	n_Pre_Intervention Group	MeanUnits_Post_Intervention Group	SDUnits_Post_Intervention Group	ost_Interven	MeanUnits_Pre_Control	SDUnits_Pre_Control	n_Pre_Control	MeanUnits_Post_Control	SDUnits_Post_Control	n_Post_Control	Pooled_SD_Pre
Abavisani et al. (2019) Part 1 Obvious Anxiety	Obvious Anxiety	-0.297	0.257	-2.18	1.848	52.74	7.41	31	49.63	7.39	31	52.48	7.27	31	51.81	7.16	31	7.34033378
Abavisani et al. (2019) Part 2 Hidden Anxiety	Hidden Anxiety	-1.380303483	0.257	-10.52	1.922	52.26	7.3	31	42.77	7.71	31	52.77	7.93	31	53.29	7.42	31	7.621512317
Akandere & Tekin (2008)	Anxiety (STAI)	-2.64	0.161432245	-16.3	0.976	55.27	6.782	30	41.93	4.2745	30	54.46	5.5	30	58.23	3.21	30	6.174363287
Asci (2003)	Trait Anxiety ONLY (STAI)	-0.312866856	0.325981724	-1.25	1.261	9.9	3.43	20	47.15	2.72	20	48.45	4.49	20	48.4	4.94	20	3.99530975
Eather et al. (2019)	Anxiety (STAI)	0.128	0.402153677	0.19	0.583	14.29	1.5	27	14.39	2.37	27	13.9	1.47	26	14.2	1.83	26	1.485369829
Erdogan Yuce & Muz (2020) Part 1 State Anxiety	State Anxiety (STAI)	-0.959	0.224	-9.79	2.254	41.31	9.54	44	32.54	9.91	44	39.46	10.82	45	42.33	11.29	45	10.20743723
Erdogan Yuce & Muz (2020) Part 2 Trait Anxiety	Trait Anxiety (STAI)	-0.419	0.174	-3.48	1.751	46.25	7.82	44	43.4	7.26	44	45.68	8.74	45	46.86	9.13	45	8.29804547
Ghorbani et al. (2014)	Anxiety	-0.974	0.349172398	-3.93	1.348	5.2	3.29	15	2.8	2.39	15	7.33	4.66	15	6.73	4.64	15	4.033590212
Ji et al. (2022) Part 1 Team Sports vs. Control	Anxiety (Self-Rating Anxiety Scale)	-0.558	0.2105	-4.916	1.5	57.23	9.3	66	46.389	8.9302	66	56.2	8.3	67	51.305	8.3645	67	8.810381628
Ji et al. (2022) Part 2 Individual Sports vs. Control	Anxiety (Self-Rating Anxiety Scale)	-0.231	0.1444	-2.143	1.625	59.14	10.2	64	49.162	10.188	64	56.2	8.3	67	51.305	8.3645	67	9.276652363
Kim et al. (2013)	Trait Anxiety	-0.804	0.122	-0.41	0.221	2.21	0.57	7	1.8	0.54	7	2.25	0.47	11	2.21	0.4	11	0.509803884
Li & Li (2017)	Anxiety (HAMA)	-0.755	0.342	-3.46	1.511	19.63	4.7	19	17.26	4.99	19	20.61	4.45	18	20.72	4.13	18	4.580276037
Li et al. (2022)	Anxiety (Self-Rating Anxiety Scale)	-1.148	0.473789767	-7.3	2.867	58.1	6.2	13	39	4.2	13	54.7	6.5	14	46.3	9.5	14	6.357766904
Ning (2020)	Anxiety (self-rating anxiety scale)	-1.467	0.287	-7.41	1.421	24.42	3.834	30	16.42	6.025	30	23.98	6.025	30	23.83	4.93	30	0.921954446
Paolucci et al. (2018) Part 1 MCT vs. Control	Anxiety (BAI)	-1.095	0.47765	-7.5	2.578	7.3	6.7	19	5.6	5	19	11.7	7	18	13.1	10	18	6.847356111
Paolucci et al. (2018) Part 2 HiiT vs. Control	Anxiety (BAI)	-0.509	0.5156	-3.8	3.033	9.1	7.9	18	9.3	8.1	18	11.7	7	18	13.1	10	18	7.46357823
Roth & Holmes (1987) Exercise vs. Control	Anxiety (STAI) reported as one measurement	-0.018	0.3478	-0.2	3	40.9	10.3	18	36.8	8.9	18	41.8	11.6	18	37	9.1	18	10.96927527
Xiao et al. (2021) Part 1 Basketball vs. Control	Anxiety (self-rating anxiety scale)	-2.016	0.2841	-10.45	1.182	52.84	5.05	33	41.84	4.43	31	54.03	5.31	34	52.59	5.04	34	5.183630002
Xiao et al. (2021) Part 2 BDJ vs. Control	Anxiety (self-rating anxiety scale)	-1.185	0.278	-6.71	1.261	51.29	6	33	45.58	5.12	31	54.03	5.31	34	52.59	5.04	34	5.660213777
Zhang et al. (2023)	Anxiety (SAS)	-0.625	0.473101504	-5.92	4.141	56.94	9.23	9	41.6	8.85	9	54.58	9.7	9	47.52	8.72	9	9.467916878
Akandere & Demir (2011)	Depression (BDI)	-0.552	0.192	-3.58	1.231	15.72	7.004	60	13.9	5.568	60	16.53	5.922	60	17.48	7.74	60	6.485603287
Balkin et al. (2007) Part 1 Aerobic vs. Control	Depression (BDI)	-0.32	0.265	-2.18	1.769	7.59	6.82	46	5.39	5.72	46	9.5	6.83	14	7.57	6.05	14	6.822242654
Balkin et al. (2007) Part 2 Anaerobic vs. Control	Depression (BDI)	-0.078	0.377	-0.57	2.667	9.24	7.67	21	7	8.65	21	9.5	6.83	14	7.57	6.05	14	7.350559039
Ghorbani et al. (2014)	Depression (GHQ)	-1.061	0.350309912	-0.42	1.304	4	1.8	15	3.91	2.39	15	5.33	5.2	15	4.33	4.45	15	3.891015292
Hermat-Far et al. (2012)	Depression (BDI)	-1.238	0.573	-6.2	2.676	25	5.3	10	16.6	6.9	10	23.8	4.7	10	22.8	4.9	10	5.008991915
Herbert et al. (2020) Exercise vs. Control Online Study Only	Depression (BDI) Online Study	-0.052	0.3358	-0.4	2.419	9.4	6.085197	19	5.9	4.336669	19	6.1	8.602196	31	6.3	6.614504	31	7.754657856
Kim et al. (2013)	Depression (BDI)	-1.019	0.536	-0.39	0.189	0.79	0.51	7	0.36	0.34	7	0.74	0.28	11	0.75	0.42	11	0.382802168
Ning (2020)	Depression (self-rating depression scale)	-1.256	0.641	-7.3	1.879	21.75	6.573	30	14.73	9.311	30	22.12	4.93	30	22.03	4.382	30	5.809872159
Paolucci et al. (2018) PART 1 MCT vs. Control	Depression (BDI)	-1.498	0.4262	-13.7	3.391	11.4	8.8	19	9.4	8.8	19	16.7	9.5	18	23.1	11.7	18	9.146693392
Paolucci et al. (2018) PART 2 HiiT vs. Control	Depression (BDI)	-1.147	0.4218	-10.9	3.155	13.2	9.5	18	12.2	6.5	18	16.7	9.5	18	23.1	11.7	18	9.5
Roth & Holmes (1987) Exercise vs. Control	Depression (BDI)	-0.135	0.2805	-0.9	1.467	7.8	6.4	18	4.1	4.5	18	7	6.9	18	5	4.3	18	6.654697589
Tomar et al. (2022)	Depression (PHQ9)	-1.111	0.381543803	-5.05	1.643	8.25	5.37	16	6.5	3.63	16	10.33	3.53	16	11.55	4.47	9	4.544106073
Yigiter & Hardee (2017)	Depression (BDI)	-1.159	0.234	-2.13	0.421	16.13	1.73	30	12.73	1.2	30	15.23	1.94	30	14.86	1.97	30	1.838001632
Zhang et al. (2023)	Depression (SDS)	-0.716	0.486503429	-8.41	5.284	69.29	12.16	9	43.39	10.17	9	57.5	11.32	9	51.8	12.16	9	11.74751037
Choi et al. (2018)	Stress (Korean version of Brief Encounter Psychosocial Instrument)	-0.048	0.261677675	-0.19	1.026	12.03	4.29	33	11.2	4.3	33	10.57	3.65	30	11.39	3.79	30	3.998531903
deVries et al. (2018)	Stress (Diener, Oishi, & Lucas (2002) 6 indicators of wellbeing)	-0.55	0.202653207	-0.49	0.4	5.7	2.11	50	5.24	1.94	49	6.6	1.88	49	5.73	2	48	1.999495039
Eather et al. (2019)	Perceived stress	-0.985539524	0.425740582	-3.51	1.48	19.52	4.19	27	14.17	5.58	27	21.9	2.76	26	17.68	5.18	26	3.561500565
Erdogan Yuce & Muz (2020)	Stress (Perceived Stress Scale)	-0.494044143	0.213663254	-4.14	1.766	27.38	7.56	44	22.43	6.94	44	26.62	9.11	45	26.57	9.49	45	8.379817846
Kim (2014)	Stress (Life Stress - wholistic score used for meta-analysis)	-3.333	0.413265306	-1	0.118	1.6	0.3	12	0.7	0.4	12	1.6	0.3	15	1.7	0.2	15	0.3
Li et al. (2015)	Stress (Chinese Percevied Stress Scale)	0.174	0.142031659	0.93	0.755	24.22	5.18	101	23.53	5.4	101	23.91	5.5	105	22.6	5.43	105	5.345531394

Paolucci et al. (2018) Part 1 MCT vs. Control	Stress (Perceived Stress Scale)	-1.028	0.42962	-7.3	2.406	18.9	7.2	19	16.6	6.6	19	22.4	7	18	23.9	8	18	7.103560475
Paolucci et al. (2018) Part 2 HiiT vs. Control	Stress (Perceived Stress Scale)	-0.634	0.44849	-4.7	2.617	16.9	7.8	18	19.2	7.7	18	22.4	7	18	23.9	8	18	7.410802925
Xiao et al. (2021) Part 1 Basketball vs. Control	Stress (Perceived Stress Scale)	-0.229	0.0585	-5.68	1.164	33.87	5	33	27	4.21	31	34.5	5.82	34	32.68	5.08	34	24.83119936
Xiao et al. (2021) Part 2 BDJ vs. Control	Stress (Perceived Stress Scale)	-0.577	0.26747	-3.16	1.173	34.23	5.1	33	29.52	4.3	31	34.5	5.82	34	32.68	5.08	34	5.477378935
Zheng et al. (2015)	Stress (Chinese Percevied Stress Scale)	-0.286	0.162264325	-1.52	0.858	23.48	5.35	95	22.99	5.65	95	24.93	5.29	103	24.51	6.36	103	5.318859975
