# Supplementary material for: Effectiveness of physical activity interventions on undergraduate students’ mental health: systematic review and meta-analysis
Source: Health Promot Int. 2024 Jun 25;39(3):daae054. doi: 10.1093/heapro/daae054 (PMC11196957; doi:10.1093/heapro/daae054)
Supplement: daae054_suppl_Supplementary [file daae054_suppl_supplementary.zip › Huang_Supplementary_E_Meta_Analysis_Descriptive_Data.pdf]

Counter	Study Author	Type of PA	Number of Participants	Frequency/Duration	Study Design	Comparator/Control	Mental Health Outcomes	Misc Outcomes	Pri Outcome Instrument	Sec Outcome	Sec Outcome Instrument	Behavioural Theory (Y/N)
1	Abavisani et al. (2019)	Pilates (8 weeks)	62 (31 PA; 31 control)	twice a week, 2 hours, 8 weeks	RCT	No exposure to Intervention (asked to resume regular activities)	1: Anxiety		Spielberger Obvious & Hidden Anxiety Inventory	N/A		N
3	Akandere & Demir (2011)	Dance (12 weeks)	120 (60 PA; 60 Control)	3 times a week, 12 weeks	RCT	No exposure to Intervention	1: Depression		Beck's Depression Inventory	N/A		N
42	Akandere & Tekin (2008)	Mixed (gymnastics, volleyball, athletics) (6 weeks)	143 (67 Yoga; 67 fitness)	7 days a week, 30 mins, 6 weeks	Non-Randomised Experimental Study	No exposure to Intervention (asked to resume regular activities)	1: Anxiety		STAI	N/A		N
6	Asic (2003)	Step Dance (10 weeks)	40 (20 PA, 20 control)	3 times a week, 50 mins, 10 weeks	RCT	No exposure to Intervention (instructed to not engage in any PA)	1: Anxiety<br>2: Physical Self-Concept<br>3: Physical Self-Esteem	Physical Self-Concept	1: State-Trait Anxiety Inventory<br>2: Physical Self-Description Questionnaire	Body Fat		N
7	Balkin et al. (2007)	Unspecified aerobic exercise & weight training (6 weeks)	46 Aerobic; 21 Anaerobic, 14 Control	6 weeks	No information provided	No exercise	1: Depression		Beck's Depression Inventory	N/A		N
22	Choi et al. (2018)	Unspecified aerobic exercise & weight (9 weeks)	33 PA; 30 Control	3 levels of intervention: individual, group, and university level.<br><br>Group level:<br>Exercise training<br>- 50 minutes every week for 9 weeks<br>Small Group Exercise Sessions<br>- 1-2 times per week outside of individual and group sessions	Non-Randomised Experimental Study	Education-only Control	1: Stress		Brief Encounter Psychosocial Instrument (BEPSI) Korean Version	1: PA<br>2: SB<br>3: Physiological Measurements<br>4: Step Count	1: IPAQ<br>2: IPAQ<br>3: Waist circumference, BMI<br>4: MI Band	YES - Nies & Kershaw's model of PA and health outcomes<br>(establishment of exercise goals, changing lifestyle to prevent relapse, improving self-efficacy and changing understandings of benefits of exercise, utilisation of social support, and increasing ease of access to exercise equipment)
11	deVries et al. (2018)	Low Intensity Running (6 weeks)	97 (49 PA, 48 control)	3 times a week, 1 hour, 6 weeks	RCT	Wait-list Control	1: Self-Efficacy<br>2: Stress		1: Psychological Detachment from studies when exercising<br>2-4 Diener, Oishi, & Lucas (2002) 6 indicators of wellbeing	N/A		N
12	Eather et al. (2019)	High-intensity Interval Training (8 weeks)	53 (27 PA; 26 control)	3 times a week, 8 mins (wk1-4), 10 mins (wk5-6), 12 mins (wk7-8), 8 weeks	RCT	Wait-list Control	1: Anxiety<br>2: Cognitive Function<br>3: Stress	Cognitive Funciton	1: STAI<br>2: Cog measures of Exc. Function<br>3: PSS	Physiological Function & Muscle Mass	1: Push Ups<br>2: Standing Jump<br>3: Skeletal Muscle Mass	Maybe? Used SAAFE (Supportive, Active, Autonomous, Fair, and Enjoyable) principles
13	Erdogan Yuce & Muz (2020)	Yoga (4 weeks)	44 PA; 45 Control	once a week, 60 mins, 4 weeks	Non-Randomised Experimental Study	No exposure to Intervention	1: Anxiety<br>2: Stress<br>3: Quality of Life<br>4: Physical Health<br>5: Psychological Health<br>6: Social relationships	Physical and Psychological health	1: STAI<br>2: PSS<br>3-6: QoL (WHOQOL-BREFTR)	N/A		N
17	Ghorbani et al. (2014)	Running & Rope Skipping (6 weeks)	30 (15 PA; 15 control)	three times a week, 60 mins, 6 weeks	RCT	No exposure to Intervention	1: Anxiety<br>2: Depression<br>3: Social Function		GHQ28	1: Vo2 Max<br>2: BMI<br>3: Physical Health<br>4: Total GHQ Score	Physiological Measures & GHQ	N
20	Herbert et al. (2020) Online Study ONLY	Cardiovascular and Muscular Endurance Exercises (6 weeks)	Online Study: 153 (30 PA; 30 writing; 31 wait)<br>Lab Study: 32 (10 PA; 11 motor; 9 wait)	2 times a week, four 8 min, four 12 min, four 16 min, 6 weeks	RCT	Wait-list Control, Expressive Writing, and Motor Coordination	1: Anxiety<br>2: Coping strategy<br>3: Depression<br>4: Quality of Life		1: STAI<br>2: Stress Coping Inventory<br>3: BDI<br>4: WHOQOL-BREF	PA	Global PA Questionnaire	N
19	Hermat-Far et al. (2012)	Running (8 weeks)	10 PA; 10 Control	3 times a week, 40-60 mins, 8 weeks	Non-Randomised Experimental Study	No exposure to Intervention (instructed to not engage in any PA)	1: Depression		BDI	Physiological Stress	Plasma Serotonin Levels	N
4A	Ji et al. (2022)	Team Sports and Individual Strength and Aerobic Training	197 (66 team sports, 64 individual, 67 control)	Twice a week, 45-50 mins, 7 weeks	RCT	No exposure to Intervention	1: Anxiety		Self-Rating Anxiety Scale	1: BMI<br>2: Body Fat<br>3: Smoking	1: Physiological Measurements<br>2: PSQI	N
24	Kim et al. (2013)	Kuok Sun Do (4 weeks)	18 (7 PA; 11 control)	10, 70 min sessions over 4 weeks	RCT	Wait-list Control	1: Anxiety<br>2: Depression<br>3: Self-Efficacy		1: STAI<br>2: BDI<br>3: General Self-Efficacy Scale (GSE Scale)	N/A		N
26	Li & Li (2017)	Running & Weight Exercises (14 weeks)	37 (19 PA; 19 control)	Preconditioning (30 min sessions, 5 times a week, over 2 weeks)<br><br>Aerobic Physical Activity (45 min sessions, 5 times a week, for 12 weeks)	RCT	No exposure to Intervention	1: Anxiety		Hamilton Anxiety Scale (HAMA)	BMI		N
28	Li et al. (2015)	Baduanjin Exercise (12 weeks)	206 (101 PA; 105 control)	1 hour per day, 5 days per week, for 12 weeks	RCT	No exposure to Intervention (asked to resume regular activities)	1: General Self-Efficacy<br>2: QOL<br>3: Self-reported Psyc Symptom Intensity<br>4: Stress	SCL-90	1: GSES<br>2: WHOQOL-BREF<br>3: SCL-90<br>4: PSS	1: PA<br>2: SB		N
3A	Li et al. (2022)	Resistance Training	27 (13 PA, 14 control)	Twice a week, 50mins, 8 weeks	RCT	No exposure to Intervention (asked to resume regular activities)	Anxiety		Self-Rating Anxiety Scale	Heart Rate Variability		N
34	Ning (2020)	Unspecified aerobic exercise (20 weeks)	60 (30 PA; 30 control)	twice a week, 120 mins, 20 weeks	RCT	No exposure to Intervention (asked to resume regular activities)	1: Anxiety<br>2: Depression		1: Self-Rating Anxiety Scale<br>2: Self-Rating Depression Scale	N/A		N
35	Paolucci et al. (2018)	High-intensity Interval Training & Moderate-Intensify Training (6 weeks)	55 (18 HIIT; 19 Mod Inten Training; 18 control)	three times a week, 20-27mins, 6 weeks	RCT	No exposure to Intervention (instructed to not engage in any PA)	1: Anxiety<br>2: Depression<br>3: Stress		1: BAI<br>2: BDI<br>3: PSS	Aerobic Fitness	Measure of V02	N
36	Roth & Holmes (1987)	Unspecified aerobic exercise (11 weeks)	55 (18 aerobic; 19 relax; 18 control)	three times a week, 30 mins, 11 weeks	RCT	No exposure to Intervention	1: Anxiety<br>2: Depression<br>3: Distress		1: STAI<br>2: BDI<br>3: Hopkins Symptooms Checklist (SCL-90)	1: Aerobic Capacity<br>2: HR	Aerobic Capacity Estimate<br>HR (bpm)	N
9A	Tomar et al. (2023)	Recreational Team Sports (12 weeks)	26 (16 PA, 10 Control)	three times a week, 30 mins, 12 weeks	Non-Randomised Experimental Study	No exposure to Intervention	1: Depression<br>2: Wellbeing		1: PHQ-9<br>2: WHO-5	1: Exercise Motivation<br>2: Self Esteem<br>3: Sleep Quality<br>4: Eating Disorder	1: Behavioural Regulation in Exercise Questionnaire (BREQ-2)<br>2: Rosenberg Self-Esteem Scale<br>3: PSQI<br>4: Eating Disorder Examination Questionnaire (EDE-Q)	N
45	Xiao et al. (2021)	Basketball and Baduanjin exercise (12 weeks)	96 (31 basketball, 31 baduanjin, 34 control)	Three times a week, 90 mins, 12 weeks	RCT	No exposure to Intervention (asked to resume regular activities)	1: Anxiety<br>2: Feelings of Inadequacy<br>3: Loneliness<br>4: Problematic Mobile Phone Use<br>5: Stress	Feelings of Indequacy, problematic phone use	1: Self-Rating Anxiety Scale<br>2: Janis & Field's Feelings of Inadequacy Scale<br>3: UCLA Loneliness<br>4: Problematic Mobile Phone Use<br>5: PSS	N/A		N

47	Yigiter & Hardee (2017)	Sports (Tennis) & Unspecified Aerobic Exercises (8 weeks)	60 (30 PA; 30 control)	three times a week, 60 mins, 8 weeks	RCT	No exposure to Intervention (instructed to not engage in any PA)	1: Depression<br>2: Self-Esteem		1: BDI<br>2: Rosenberg Self-Esteem Scale		N/A		N
7A	Zhang et al. (2023)	Tai Chi Quan (8 weeks)	18 (9 PA, 9 Control)	fives times a week, 60 mins, 8 weeks	RCT	No exposure to Intervention (asked to resume regular activities)	1: Anxiety<br>2: Depression		1: Self-Rating Anxiety Scale<br>2: Self-Rating Depression Scale	fMRI			N
50	Zheng et al. (2015)	Tai Chi Quan (12 weeks)	195 (92 PA; 103 control)	five times a week, 60 mins, 12 weeks	RCT	No exposure to Intervention (asked to resume regular activities)	1: Psychological Symptoms<br>2: QoL<br>3: Self-Efficacy<br>4: Stress	SCL-90	1: SCL-90<br>2: WHOQOL-BREF<br>3: GSES<br>4: PSS		1: PA<br>2: SB	PA/SB	N
