# Supplementary material for: Effectiveness of physical activity interventions on undergraduate students’ mental health: systematic review and meta-analysis
Source: Health Promot Int. 2024 Jun 25;39(3):daae054. doi: 10.1093/heapro/daae054 (PMC11196957; doi:10.1093/heapro/daae054)
Supplement: daae054_suppl_Supplementary [file daae054_suppl_supplementary.zip › Huang_Supplementary_F_Meta_Output.docx]

| Meta-Analyses Output | | | | | | | | | | |
| --- | --- | --- | --- | --- | --- | --- | --- | --- | --- | --- |
| Outcome | N interventions | I^2 | Q | p-Q | Effect (Random Effects) | Lower CI | Upper CI | p-value | Begg’s z score | Begg’s p-value |
| Anxiety | 20 | 90.28676 | 195.6094 | 0.00 | -0.88 | -1.23 | -0.52 | 0.00 | -0.32461 | 0.795102 |
| Depression | 14 | 49.65872 | 25.82374 | 0.00 | -0.73 | -1.00 | -0.47 | 0.00 | -0.87592 | 0.443421 |
| Stress (with Kim) | 11 | 86.969 | 76.74007 | 0.00 | -0.61 | -0.94 | -0.28 | 0.000259 | -2.1798 | 0.04296 |
| Stress (w/o Kim) | 10 | 58.468 | 21.67000 | 0.009986 | -0.34 | -0.53 | -0.14 | 0.000644 | -1.96774 | 0.073638 |

| Trim-and-fill Analyses | | | | | |
| --- | --- | --- | --- | --- | --- |
| Outcome | N-Total-DL | Est-TrimFill-DL | LCL-TrimFill-DL | UCL-TrimFill-DL | TrimFill-Side-DL |
| Stress w Kim | 11 | -0.61422 | -0.94374 | -0.2847 | right |
| Stress w/o Kim (2014) | 10 | -0.25966 | -0.4515 | -0.06781 | right |
