# Supplementary material for: Effectiveness of physical activity interventions on undergraduate students’ mental health: systematic review and meta-analysis
Source: Health Promot Int. 2024 Jun 25;39(3):daae054. doi: 10.1093/heapro/daae054 (PMC11196957; doi:10.1093/heapro/daae054)
Supplement: daae054_suppl_Supplementary [file daae054_suppl_supplementary.zip › Huang_Supplementary_C_Review_References.doc]

**Supplementary E: Review References**

1. Abavisani, M., Lakzian, R., Sarchahi, Z., Jafariani, S., & Pourjafar, H. (2019). the Effect of Pilates Exercise on Anxiety in Students of Department of Medical Emergency- a Clinical Trial. *Journal of Evolution of Medical and Dental Sciences*, *8*(21), 1755–1758. https://doi.org/10.14260/jemds/2019/385
2. Ahmed, N., Mehmood, N., & Nizami, G. N. (2021). Pre and Post Analysis of Stress Reduction Following Pilates Exercise among Undergraduate Students of Physical Therapy. *Medical Forum Monthly*, *32*(10), 224–227.
3. Akandere, M., & Demir, B. (2011). The effect of dance over depression. *Collegium Antropologicum*, *35*(3), 651–656. http://www.ncbi.nlm.nih.gov/pubmed/22053537
4. Akandere, M., & Tekin, A. (2008). The Effect of Physical Exercise on Anxiety. *The Sport Journal*, *24*.
5. Alsaraireh, F. A., & Aloush, S. M. (2017). Mindfulness meditation versus physical exercise in the management of depression among nursing students. *Journal of Nursing Education*, *56*(10), 599–604. https://doi.org/10.3928/01484834-20170918-04
6. Arbinaga, F., Fernández-Ozcorta, E., Sáenz-López, P., & Carmona, J. (2018). The psychological effects of physical exercise: A controlled study of the placebo effect. *Scandinavian Journal of Psychology*, *59*(6), 644–652. https://doi.org/10.1111/sjop.12482
7. Aşçi, F. H. (2003). The effects of physical fitness training on trait anxiety and physical self-concept of female university students. *Psychology of Sport and Exercise*, *4*(3), 255–264. https://doi.org/10.1016/S1469-0292(02)00009-2
8. Barğı, G. (2022). Effectiveness of Physical Activity Counseling in University Students Educated by Distance Learning During COVID-19 Pandemic: A Randomized-Controlled Trial. *Journal of Basic and Clinical Health Sciences*, *6*(2), 374–384. https://doi.org/10.30621/jbachs.1027410
9. Balkin, R. S., Tietjen-Smith, T., Caldwell, C., & Shen, Y.-P. (2007). The Utilization of Exercise to Decrease Depressive Symptoms in Young Adult Women. *Adultspan Journal*, *6*(1), 30–35. https://doi.org/10.1002/j.2161-0029.2007.tb00027.x
10. Berger, B. G., & Owen, D. R. (1987). Anxiety reduction with swimming: Relationships between exercise and state, trait, and somatic anxiety. *International Journal of Sport Psychology.Vol 18(4)*, *October*, 286–302.
11. Biber, D. D., & Knoll, C. (2020). Exercise Is Medicine on Campus®: A Pilot Study. *Recreational Sports Journal*, *44*(2), 149–157. https://doi.org/10.1177/1558866120964815
12. Caldwell, K., Emery, L., Harrison, M., & Greeson, J. (2011). Changes in mindfulness, well-being, and sleep quality in college students through taijiquan courses: A cohort control study. *Journal of Alternative and Complementary Medicine*, *17*(10), 931–938. https://doi.org/10.1089/acm.2010.0645
13. Choi, J. Y., Chang, A. K., & Choi, E. J. (2018). Effects of a physical activity and sedentary behavior program on activity levels, stress, body size, and sleep in sedentary Korean college students. *Holistic Nursing Practice*, *32*(6), 287–295. https://doi.org/10.1097/HNP.0000000000000282
14. Deng, C., Yu, Q., Luo, G., & Lu, S. (2022). Effects of 16 Weeks of Cheerleading on Physical Self-Esteem and Mental Health of Female College Students. *Frontiers in Psychology*, *13*(June), 1–8. https://doi.org/10.3389/fpsyg.2022.925162
15. de Vries, J. D., van Hooff, M. L. M., Geurts, S. A. E., & Kompier, M. A. J. (2018). Trajectories of well-being during an exercise randomized controlled trial: The role of exposure and exercise experiences. *Stress and Health*, *34*(1), 24–35. https://doi.org/10.1002/smi.2758
16. Eather, N., Riley, N., Miller, A., Smith, V., Poole, A., Vincze, L., Morgan, P. J., & Lubans, D. R. (2019). Efficacy and feasibility of HIIT training for university students: The Uni-HIIT RCT. *Journal of Science and Medicine in Sport*, *22*(5), 596–601. https://doi.org/10.1016/j.jsams.2018.11.016
17. Erdoğan Yüce, G., & Muz, G. (2020). Effect of yoga-based physical activity on perceived stress, anxiety, and quality of life in young adults. *Perspectives in Psychiatric Care*, *56*(3), 697–704. https://doi.org/10.1111/ppc.12484
18. Forseth, B., Polfuss, M., Brondino, M., Hunter, S. D., Lawlor, M. W., Beatka, M. J., Prom, M. J., Eells, J., & Lyons, J. A. (2022). Adherence to and changes in mental and physiological health during an 8-week yoga intervention: A pilot study. *Journal of Bodywork and Movement Therapies*, *30*, 203–209. https://doi.org/10.1016/j.jbmt.2022.01.004
19. Fukui, K., Suzuki, Y., Kaneda, K., Kuroda, S., Komiya, M., Maeda, N., & Urabe, Y. (2021). Do “stay-at-home exercise” videos induce behavioral changes in college students? A randomized controlled trial. *Sustainability (Switzerland)*, *13*(21), 1–11. https://doi.org/10.3390/su132111600
20. Gaskins, R., Jennings, E., Thind, H., Becker, B., & Bock, B. (2014). Acute and Cumulative Effects of Vinyasa Yoga on Affect and Stress among College Students Participating in an Eight-week Yoga Program: A Pilot Study. *International Journal of Yoga Therapy*, *24*(1), 63–70. https://doi.org/10.17761/ijyt.24.1.l8466h29060x13vq
21. Ghorbani, F., Heidarimoghadam, R., Karami, M., Fathi, K., Minasian, V., & Bahram, M. E. (2014). The effect of six-week aerobic training program on cardiovascular fit-ness, body composition and mental health among female students. *Journal of Research in Health Sciences*, *14*(4), 264–267.
22. Hamed, N. S., Abdel-aziem, A. A., Muhsen, B. A., Eid, M. M., Allam, H. A., El-Gendy, A. M., & El Sayyad, L. K. (2021). The effect of aerobic training versus cognitive behavioral therapy in management of anxiety, depression and stress related to COVID 19 pandemic among university students: A randomized controlled trial. *MEDICAL SCIENCE*, *25*(115), 2233–2246.
23. Hemat-Far, A., Shahsavari, A., & Mousavi, S. R. (2007). Effects of Selected Aerobic Exercises on the Depression and Concentrations of Plasma Serotonin in the Depressed Female Students Aged 18 to 25. *The Journal of Applied Research*, *12*(1), 47–52.
24. Herbert, C., Meixner, F., Wiebking, C., & Gilg, V. (2020). Regular physical activity, short-term exercise, mental health, and well-being among university students: the results of an online and a laboratory study. *Frontiers in psychology*, *11*, 509.
25. Huang, G. (2021). The effect of aerobic exercise on the mental health of college students. *Journal of Sport Psychology*, *30*(4), 132–139.
26. Ji, C., Yang, J., Lin, L., & Chen, S. (2022). Anxiety and Sleep Quality Amelioration in College Students: A Comparative Study between Team Sports and Individual Sports. *Behavioral Sciences*, *12*(5). https://doi.org/10.3390/bs12050149
27. Johnston, S. A., Roskowski, C., He, Z., Kong, L., & Chen, W. (2021). Effects of team sports on anxiety, depression, perceived stress, and sleep quality in college students. *Journal of American College Health*, *69*(7), 791–797. https://doi.org/10.1080/07448481.2019.1707836
28. Kim, J. H., Yang, H., & Schroeppel, S. (2013). A pilot study examining the effects of Kouk Sun Do on university students with anxiety symptoms. *Stress and Health*, *29*(2), 99–107. https://doi.org/10.1002/smi.2431
29. Kim, S. D. (2014). Effects of yogic exercises on life stress and blood glucose levels in nursing students. *Journal of Physical Therapy Science*, *26*(12), 2003–2006. https://doi.org/10.1589/jpts.26.2003
30. Li, L., & Liu, X. (2013). Experimental Studies of Physical Exercises to Improve Students Sleep Quality and Mental Health. *Advances in Psychology Research*, *98*(8).
31. Li, H., & Li, P. (2017). Effects of Aerobic Exercise on Female Obese Students with Anxiety Disorders. In *2017 7Th Ese International Conference on Sports, Health and Management, 73,* 51–56. https://doi.org/10.26602/lnms.2017.73.51
32. Li, M., Fang, Q., Li, J., Zheng, X., Tao, J., Yan, X., Lin, Q., Lan, X., Chen, B., Zheng, G., & Chen, L. (2015). The effect of Chinese traditional exercise-Baduanjin on physical and psychological well-being of college students: A randomized controlled trial. *PLoS ONE*, *10*(7), 1–16. https://doi.org/10.1371/journal.pone.0130544
33. Li, R., Yan, R., Cheng, W., & Ren, H. (2022). Effect of resistance training on heart rate variability of anxious female college students. *Frontiers in Public Health*, *10*(1). https://doi.org/10.3389/fpubh.2022.1050469
34. Mailey, E. L., Wójcicki, T. R., Motl, R. W., Hu, L., Strauser, D. R., Collins, K. D., & McAuley, E. (2010). Internet-delivered physical activity intervention for college students with mental health disorders: a randomized pilot trial. *Psychology, Health & Medicine*, *15*(6), 646-659.
35. Margulis, A., Andrews, K., He, Z., & Chen, W. (2021). The effects of different types of physical activities on stress and anxiety in college students. *Current Psychology*. https://doi.org/10.1007/s12144-021-01881-7
36. McCann, I. L., & Holmes, D. S. (1984). Influence of aerobic exercise on depression. *Journal of Personality and Social Psychology*, *46*(5), 1142–1147. https://doi.org/10.1037/0022-3514.46.5.1142
37. McFadden, T., Fortier, M. S., & Guérin, E. (2017). Investigating the effects of Physical Activity Counselling on depressive symptoms and physical activity in female undergraduate students with depression: A multiple baseline single-subject design. *Mental Health and Physical Activity*, *12*, 25–36. https://doi.org/10.1016/j.mhpa.2017.01.002
38. Murray, A., Marenus, M., Cahuas, A., Friedman, K., Ottensoser, H., Kumaravel, V., Sanowski, J., & Chen, W. (2022). The Impact of Web-Based Physical Activity Interventions on Depression and Anxiety Among College Students: Randomized Experimental Trial. *JMIR Formative Research*, *6*(4). https://doi.org/10.2196/31839
39. Ning, G. (2020). Influence of aerobics on modulation of depression and anxiety among college students. *Revista Argentina de Clinica Psicologica*, *29*(1), 662–667. https://doi.org/10.24205/03276716.2020.88
40. Paolucci, E. M., Loukov, D., Bowdish, D. M. E., & Heisz, J. J. (2018). Exercise reduces depression and inflammation but intensity matters. *Biological Psychology*, *133*(January), 79–84. https://doi.org/10.1016/j.biopsycho.2018.01.015
41. Roth, D. L., & Holmes, D. S. (1987). Influence of aerobic exercise training and relaxation training on physical and psychologic health following stressful life events. *Psychosomatic Medicine*, *49*(4), 355–365. https://doi.org/10.1097/00006842-198707000-00004
42. Sabourin, B. C., Watt, M. C., Krigolson, O. E., & Stewart, S. H. (2016). Two interventions decrease anxiety sensitivity among high anxiety sensitive women: Could physical exercise be the key? *Journal of Cognitive Psychotherapy*, *30*(2), 131–146. https://doi.org/10.1891/0889-8391.30.2.131
43. Salehian, M. H., Talab, A. H., & Ghanati, P. (2021). Effectiveness of Qigong exercises and resilience training on the perceived stress of male students due to COVID-19. *Journal of Psychopathology*, *27*(4), 204–211. https://doi.org/10.36148/2284-0249-428
44. Schmalzl, L., Powers, C., Zanesco, A. P., Yetz, N., Groessl, E. J., & Saron, C. D. (2018). The effect of movement-focused and breath-focused yoga practice on stress parameters and sustained attention: A randomized controlled pilot study. *Consciousness and Cognition*, *65*(October 2017), 109–125. https://doi.org/10.1016/j.concog.2018.07.012
45. Severtsen, B., & Bruya, M. A. (1986). Effects of meditation and aerobic exercise on EEG patterns. In *The Journal of neuroscience nursing : journal of the American Association of Neuroscience Nurses* (Vol. 18, Issue 4, pp. 206–210). https://doi.org/10.1097/01376517-198608000-00010
46. Sharp, P., & Caperchione, C. (2016). The effects of a pedometer-based intervention on first-year university students: A randomized control trial. *Journal of American College Health*, *64*(8), 630–638. https://doi.org/10.1080/07448481.2016.1217538
47. Sun, Y., Zhang, B., Ji, A., & Sun, W. (2022). A Study on the Impact of Wushu Sports Health on College Students’ Mental Health. *Journal of Environmental and Public Health*, *2022*. https://doi.org/10.1155/2022/5841017
48. Terzioğlu, Z. A., Çakır-Çelebi, S. G., & Yıldız, M. (2022). Effect of online mindfulness-based physical exercise program on psychological well-being and life satisfaction during the Covid-19 pandemic. *Current Psychology*, *1989*. https://doi.org/10.1007/s12144-022-04205-5
49. Tomar, R., Surujlal, J., & Allen, J. A. (2023). Mixed Recreational Team Games Can Improve the Psychological Health in University Youth Post-COVID-19. *International Journal of Human Movement and Sports Sciences*, *11*(4), 691–698. https://doi.org/10.13189/saj.2023.110401
50. Tong, J., Qi, X., He, Z., Chen, S., Pedersen, S. J., Cooley, P. D., Spencer-Rodgers, J., He, S., & Zhu, X. (2021). The immediate and durable effects of yoga and physical fitness exercises on stress. *Journal of American College Health*, *69*(6), 675–683. https://doi.org/10.1080/07448481.2019.1705840
51. Tucker, L. A., & Maxwell, K. (1992). Effects of weight training on the emotional well-being and body image of females: Predictors of greatest benefit. In *American Journal of Health Promotion* (Vol. 6, Issue 5, pp. 338–344). https://doi.org/10.4278/0890-1171-6.5.338
52. Wang, Y., Jia, N., Zhou, Y., Fu, L., Fan, L., & Li, B. (2023). A comparison of the effects of remote coaching HIIT training and combined exercise training on the physical and mental health of university students. *Frontiers in Psychology*, *14*. https://doi.org/10.3389/fpsyg.2023.1182332
53. Xiao, T., Jiao, C., Yao, J., Yang, L., Zhang, Y., Liu, S., Grabovac, I., Yu, Q., Kong, Z., Yu, J. J., & Zhang, J. (2021). Effects of Basketball and Baduanjin Exercise Interventions on Problematic Smartphone Use and Mental Health among College Students: A Randomized Controlled Trial. *Evidence-Based Complementary and Alternative Medicine*, *2021*. https://doi.org/10.1155/2021/8880716
54. Yazici, A. B., Gul, M., Yazici, E., & Gul, G. K. (2016). Tennis enhances well-being in university students. *Mental Illness*, *8*(1), 20–22. https://doi.org/10.4081/mi.2016.6510
55. Yigiter, K., & Hardee, J. T. (2017). Decreasing Depression By Improving the Level of Self-Esteem in a Tennis Training Program for Female University Students. *Asean Journal of Psychiatry*, *18*(1), 61–70.
56. Zhang, H., & Luo, S. (2020). Promoting effects of water sports on the mental health of college students. *Revista Argentina de Clinica Psicologica*, *29*(2), 577–583. https://doi.org/10.24205/03276716.2020.280
57. Zhang, J., Gao, T., Li, Y., Song, Z., Cui, M., Wei, Q., Chen, Z., Peng, F., & Lyu, S. (2023). The effect of Bafa Wubu of Tai Chi on college students’ anxiety and depression: A randomized, controlled pilot study. *Frontiers in Physiology*, *14*(January), 1–10. https://doi.org/10.3389/fphys.2023.1036010
58. Zheng, G., Lan, X., Li, M., Ling, K., Lin, H., Chen, L., Tao, J., Li, J., Zheng, X., Chen, B., & Fang, Q. (2015). Effectiveness of Tai Chi on physical and psychological health of college students: Results of a randomized controlled trial. *PLoS ONE*, *10*(7), 1–14. https://doi.org/10.1371/journal.pone.0132605
59. Zheng, C., & Ji, H. (2021). Analysis of the intervention effect and self-satisfaction of sports dance exercise on the psychological stress of college students. *Work*, *69*(2), 637–649. https://doi.org/10.3233/WOR-213505
